# Elimination of Dog-Mediated Human Rabies Deaths by 2030: Needs Assessment and Alternatives for Progress Based on Dog Vaccination

**DOI:** 10.3389/fvets.2017.00009

**Published:** 2017-02-10

**Authors:** Ryan M. Wallace, Eduardo A. Undurraga, Jesse D. Blanton, Julie Cleaton, Richard Franka

**Affiliations:** ^1^National Center for Emerging and Zoonotic Infectious Diseases, Centers for Disease Control and Prevention, Atlanta, GA, USA

**Keywords:** infectious disease, rabies control and prevention, dog vaccination, population management, zoonotic diseases, one health, global health, rabies elimination

## Abstract

**Background:**

Rabies imposes a substantial burden to about half of the world population. The World Health Organization (WHO), World Organization for Animal Health, and the Food and Agriculture Organization have set the goal of eliminating dog-mediated human rabies deaths by 2030. This could be achieved largely by massive administration of post-exposure prophylaxis—in perpetuity—, through elimination of dog rabies, or combining both. Here, we focused on the resources needed for the elimination of dog rabies virus by 2030.

**Materials and methods:**

Drawing from multiple datasets, including national dog vaccination campaigns, rabies literature, and expert opinion, we developed a model considering country-specific current dog vaccination capacity to estimate the years and resources required to achieve dog rabies elimination by 2030. Resources were determined based on four factors: (a) country development status, (b) dog vaccination costs, (c) dog rabies vaccine availability, and (d) existing animal health workers. Our calculations were based on the WHO’s estimate that vaccinating 70% of the dog population for seven consecutive years would eliminate rabies.

**Findings:**

If dog rabies vaccine production remains at 2015 levels, we estimate that there will be a cumulative shortage of about 7.5 billion doses to meet expected demand to achieve dog rabies elimination. We estimated a present cost of $6,300 million to eliminate dog rabies in all endemic countries, equivalent to a $3,900 million gap compared to current spending. To eliminate dog rabies, the vaccination workforce may suffice if all public health veterinarians in endemic countries were to dedicate 3 months each year to dog rabies vaccination. We discuss implications of potential technology improvements, including population management, vaccine price reduction, and increases in dog-vaccinating capacities.

**Conclusion:**

Our results highlight the resources needed to achieve elimination of dog-mediated human rabies deaths by 2030. As exemplified by multiple successful disease elimination efforts, one size does not fit all. We suggest pragmatic and feasible options toward global dog rabies elimination by 2030, while identifying several benefits and drawbacks of specific approaches. We hope that these results help stimulate and inform a necessary discussion on global and regional strategic planning, resource mobilization, and continuous execution of rabies virus elimination.

…it is villainous that our pounds should be so little patronized and such swarms of dogs allowed to run unmuzzled….[Dogs] swarm in all the streets, obstruct the pavements, make night hideous with their howls… New York Daily Times (1)

## Introduction

The control of dog rabies presents a unique and challenging undertaking. The One-Health nature of rabies—requiring the integration of medical and veterinary sectors—has challenged how animal control and rabies prevention efforts are designed and implemented. Animal control has posed a challenge for centuries (1, 2), as suggested by the epigraph, which continues to resemble many urban and peri-urban settings today. Rabies is a zoonotic disease that kills an estimated 59,000 people and hundreds of thousands of animals annually, with most of the burden falling in low- and middle-income countries, particularly among children and poor urban and rural communities (3, 4). About 99% of rabies human cases originate by rabid domestic dogs (4–7). Few other zoonotic diseases have provoked the same sense of terror in humans as rabies, and dog bites in general have been a focal issue in the control of dog populations (1, 8). Controlling dog rabies substantially reduces human exposures (9, 10) and can be accomplished through periodic campaigns of dog vaccination (9, 11, 12). World Health Organization (WHO) recommends recurrent vaccination campaigns covering at least 70% of the dog population to control and potentially eliminate dog rabies (7, 13, 14).

The One-Health character of rabies has for centuries illuminated inherent divisions of responsibilities between institutions focused on animal health and human health. This has often complicated and delayed the development of modern rabies control programs, as most early efforts to control rabies were relinquished to non-public health agencies (1). As public health agencies became more involved in rabies prevention, particularly after the introduction of post-exposure prophylaxis, they remained primarily reactive, providing care to humans after a potential rabies exposure (2). Even today, determining whether health or agriculture has responsibility for leading control efforts often impedes initiation of rabies control plans (7).

Despite these challenges, dog rabies has been successfully eliminated in most of the Western Hemisphere, western Europe, and some Asian countries. Effective control of dog rabies at the community level through dog control programs (e.g., confinement, stringent leash-law legislation, and destruction of stray animals) began in parts of Europe as early as the nineteenth century (2). However, mass vaccination programs starting in the 1920s and 1930s were largely responsible for the elimination of dog rabies in Canada, Europe, Japan, and the United States (2, 15–18). Similarly, regional rabies control efforts in Latin America, with support from the Pan American Health Organization, were initiated in the early 1980s and have been successful in reducing the number of reported cases of rabies in dogs by 98% (19–21). These success stories have resulted from a combined effort involving mass dog vaccination, dog population control, and coordination at national and community levels, all supported and promoted by national governments (2, 22, 23).

In 2016, the WHO, the World Organization for Animal Health (OIE), the Food and Agriculture Organization (FAO), and many non-governmental organizations agreed to mobilize their member states, portfolios, and resources to eliminate dog-mediated human rabies by 2030 (24, 25). Many dog-rabies endemic countries (DECs) are at the early stages of their control efforts and are still overcoming barriers related to limited understanding of the local epidemiology, logistic and operational challenges, lack of resources, and competing priorities of other diseases (3, 26, 27). Many of these barriers have been addressed and overcome by other nations, affording the opportunity for those countries still at early stages to benefit from prior experiences (4, 6, 26, 28).

This analysis intends to provide both a realistic assessment and feasible projections for a path toward global dog rabies elimination by 2030 through dog vaccination. Building on previous experience of dog vaccination programs (7, 19, 21, 29), we focused on four factors that are likely to affect a country’s ability to eliminate dog rabies: country development, cost of vaccinating 70% of the dog population, dog rabies vaccine production, and availability of trained personnel for vaccine administration. We considered and evaluated several categories of plausible technological improvements and innovations as a sensitivity analysis, including increasing vaccination capacity, decreasing vaccine costs, and massive dog population management efforts. Our results quantitatively highlight some of the main challenges and provide overview of possible modifications, directions, and pathways to be considered. Our goal is to provide a realistic description of the *status quo* and present gaps hampering possible elimination. In particular, this manuscript highlights the urgent need to initiate action among public health officers, donors, academics, and the global rabies community if 2030 is to be maintained as a realistic target for elimination.

## Materials and Methods

### Overview

We combined multiple data sources to derive quantitative estimates of the four factors that are likely to influence the feasibility of dog rabies elimination by means of dog vaccination: country development, cost of dog vaccination programs, potential demand for dog vaccine, and the current number of trained dog vaccination personnel (e.g., para-veterinarians). We included a total of 192 countries in our analysis. We classified countries as either DECs or dog rabies-free countries and used United Nations (UN) geographic regions to group them to summarize our main results (30). For the purposes of this study, we assumed constant human, dog, and health-care worker populations for the time period 2017–2030.

### Framework for Eliminating Dog Rabies

We developed a theoretical global dog rabies elimination pathway (GDREP) consisting of a 13-year time frame; with the assumption that this would be enough time for even the least-developed rabies control programs to achieve elimination by 2030 if they fully committed to this achievement. A country’s starting point within the GDREP timeframe was dependent upon their current (2015) estimated dog vaccination rate, and each country was assumed to progress through the program in annual increments. We assumed that all DECs would start the program at the same time (January 1, 2017), and that countries would progress through each annual stage of the program without regress.

**Figure 1:**
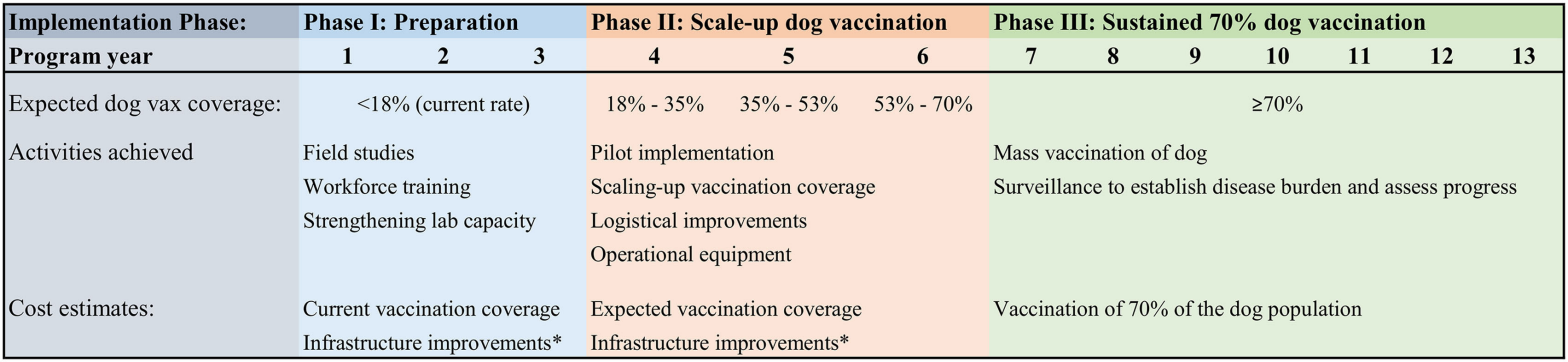
**Global Dog Rabies Elimination Pathway (GDREP): phases for a dog rabies elimination program based on 70% dog population vaccination coverage**. Notes: there is variation between and within countries for the implementation and scaling-up of national dog vaccination campaigns. Based on expert opinion from dog vaccine implementation strategies in Haiti, Ethiopia, United States, Vietnam, and Latin America, we assumed that the current dog rabies vaccination coverage was directly correlated to the number of years it will take to achieve elimination, as illustrated by the three distinct phases shown in the figure. For countries in Phase II, we estimated dog vaccination as the median value in the corresponding year range (e.g., we used 26% vaccination coverage in the rage 18–35%), or the current country vaccination rate for those countries that were already vaccinating dogs at a rate equivalent to Phase II. *Infrastructural improvement costs were estimated to be equivalent to the cost of vaccination for 10% of the country’s unvaccinated dog population.

Dog vaccination programs typically rely on robust logistical support, including infrastructure and human capital (31–34). Based on expert opinion from dog vaccine implementation strategies in Haiti, Ethiopia, United States, Vietnam, and Latin America, we assumed that the current dog rabies vaccination coverage was an indicator of a country’s capabilities to conduct mass dog vaccination campaigns. We divided the GDREP into three phases (Figure 1), as a function of their current dog vaccination rates. In this framework, a countries would progressively move toward phase III, a threshold at which they would conduct a full-scale dog vaccination campaign.

Countries with current dog vaccination coverage below 18% (i.e., 25% of WHO’s goal of 70% of the dog population vaccinated) were classified as Phase I. These countries entered the program at year 1, and we estimated that they would need the full 13 years to achieve elimination. Phase I countries were given 3 years to develop field studies, workforce training, adequate legislation, and other infrastructural improvements. We estimated the annual cost for each of the 3 years that these countries remained in Phase I as the country-specific cost of the 2015 dog vaccination efforts plus the cost for these infrastructural developments. Because we found no available evidence of the costs to scale-up a fledgling rabies vaccination program, and the exact amount is likely to vary from country to country based on available capabilities and dog population size, we defined the costs of infrastructural developments as approximately the cost to vaccinate 10% of the dog population that remained to be vaccinated. The 10% was estimated based on CDC’s dog vaccination support activities in Haiti (27, 35, 36).

Countries with vaccination coverage above 18% and below 70% were classified as Phase II. Phase II was equally divided into three 1-year periods based on current dog vaccination: 18–35% coverage countries entered the program at year 4, 36–53% coverage countries entered at year 5, and 53–69% coverage countries entered the program at year 6. Phase II countries were charged an annual rabies elimination cost equal to their current dog vaccination efforts plus the cost to vaccinate an additional 10% of their dog population. These additional costs were estimated to be necessary to further develop their mass dog vaccination programs, focused on scale-up of services, logistical improvements, and pilot implementation of elimination programs. Depending on the estimated 2015 dog vaccination rates, countries entering the program in Phase II could take 8–10 years to achieve elimination.

Countries with vaccination coverage currently at or above 70% were classified as Phase III. Based on WHO recommendations, we estimated that countries would have to vaccinate 70% of the dog population for 7 years to eliminate dog rabies (7, 13, 14). All DECs currently vaccinating at 70% entered the program at year 7, and required 7 years to achieve elimination. Therefore, if all DECs committed to dog rabies elimination in 2017 and progressed through this program, the earliest a country could achieve elimination is 7 years and the longest it would take is 13 years.

### Analysis and Input Data

Country-specific estimates for each of the four factors evaluated in this study (i.e., country development, cost of dog vaccination to achieve goal, expected number of dog vaccines required, and currently trained personnel) were derived and then aggregated by UN Regions and at the global level. Table 1 shows the main parameters and sources of data used to obtain the estimates in this study. We obtained country populations and proportion residing in urban areas for the year 2015 from the World Bank (37).

**Table 1:** **Main parameters and data sources to inform the estimates for Global Dog Rabies Elimination Pathway (GDREP), 2017–2030**.

Parameter	Value	Source
**Demographic and epidemiological data**		
Human population	Country specific	(37)
Urban population (%)	Country specific	(37)
Human-to-dog ratios (humans per dog)	Mean: 10.8	Estimated
Asia and Oceania	Urban: 7.5; rural: 14.3	(6)
China	Urban: 48.3; rural: 48.3	(6)
Africa	Urban: 21.2; rural: 7.4	(6)
The Americas	Urban: 7.5; rural: 7.5	(19)
Europe	Urban: 6.5; rural: 6.5	Estimate
HDI	Country-specific	(38)
**Dog vaccine administration**		
Vaccination coverage needed	70%	(7, 13, 14)
Vaccinated dogs by country	Country specific	(4)
Daily vaccination capacity^a^	100 dogs/day/person	(39)
Animal health workers		
Public health veterinarians	Country specific	(40)
Public health para-veterinarians	Country specific	(40)
**Cost to vaccinate^b^**		
Cost per dog vaccinated (point estimate)	$2.18	(41–43)
Vaccine, syringes, and needles (%)	26.8	(41, 42, 44)
Personnel (%)	28.5	(41, 42, 44)
Overhead and other costs (%)	44.7	(41, 42, 44)
Discounting rate (%)	3	(45)
**Dog population management^c^**		
Share of female dogs sterilized in first round (%)	70	(46)
Maintenance sterilization (%)	30	(46, 47)
Reduction in dog population over 5 years (%)	40	(47, 49)
Cost per female dog sterilized	$8.00	(47, 49)

*HDI, human development index; it is a composite measure of health, education, and income used by the United Nations Development Program (38)*.

*^a^Dog vaccination capacity is consistent with unpublished data collected by the authors of this analysis in urban, semi-urban, and rural areas of Haiti*.

*^b^All costs were adjusted to 2015 US dollars using gross domestic product implicit price deflators (48)*.

*^c^The dog population management scenario is based on the expected/plausible technological developments in coming years*.

We estimated country-specific dog populations by applying regional human-to-dog ratios to each country’s human population based on previous literature (6, 19) (Table 1). We calculated dog populations for both urban and rural areas, and the total was represented as the summation of these two values. We obtained estimates of dog rabies vaccination coverage from Hampson et al. (4).

We estimated country-specific veterinary workforce using data from OIE (40), based on two optimistic scenarios: (A) all public health veterinary workforce is available to provide dog rabies vaccinations (including private and public practice) and (B) that all public health para-veterinarian workforce is available to provide dog rabies vaccinations. For each scenario, we calculated the potential gap or surplus in vaccinator capacity for programs that operate both 1- and 3-month vaccination campaign duration. We calculated the capacity by country and then aggregated it globally. Information on veterinary workforce is voluntarily provided by countries. We imputed the size of the animal health workforce, weighted by country cluster, for countries for which data were not available.

The cost to vaccinate a dog is variable and may differ from country to country. We estimated the cost to vaccinate a dog based on the average inflation-adjusted estimate from articles of dog vaccination costs to prevent rabies in three developing countries: Chad (41), Tanzania (42), and the Philippines (43). These costs included vaccine costs (e.g., syringes, certificates, vaccine), equipment, cold chain, dog vaccinators’ salaries, transportation, awareness, and information, among others, but the specific items included varied by study. We used other published estimates to inform our sensitivity analysis on costs per vaccine (6, 44, 49). We focused solely on dog vaccination; we did not include other costs associated with rabies control, such as rabies surveillance, laboratory diagnostics, or training dog vaccination personnel, which despite being integral to an effective rabies control program cannot be readily quantified and are unlikely to change the overall conclusions of our analysis. We used a 3% discount rate in all our estimates (45), and adjusted all cost to 2015 US dollars using US gross domestic product implicit price deflators (48).

Dog rabies vaccine production is a potentially limiting factor in the effort to eliminate dog rabies globally. The 2015 estimated number of dog rabies vaccines used in 2015 was obtained from Hampson et al. (4). This current DEC dog rabies vaccine demand was assumed to approximate the supply, as there have been no reports of large-scale expiration of animal rabies vaccines. We compared the current supply of vaccine to DECs to the anticipated annual demand from 2017 to 2030 and calculated the gap in current supply to anticipated demand.

Last, we ranked the UN Regions in terms of likelihood of achieving dog rabies elimination based on aggregate indicators of the four factors used in our country-level analysis, plus two regional indicators. Criteria included: country development index, estimated funding gap for elimination, current dog vaccination coverage, gap in vaccinator capacity, proportion of the cluster that was considered rabies free, and expected years to achieve elimination. Each of the six criteria was ordered from most optimal to least optimal value and received the corresponding numerical score. We summed the country cluster rank scores to obtain a cumulative rabies elimination rank score. Lower scores represent UN Regions that appear to be more favorably situated to achieve the rabies elimination goal.

### Sensitivity Analysis, Main Assumptions, and Robustness Checks

To assess what future efforts might have the greatest impact on global dog rabies elimination, we developed four hypothetical scenarios assuming that a new technology would be developed which would impact the feasibility of dog vaccination. These illustrative scenarios included a 50% decrease in the price of dog rabies vaccine (including syringe and needle), 50% and 100% increases in the daily vaccination capacity of a vaccinator, and a global reduction in the dog population to a level that is presumed to be sustainable under currently available resources (a 40% reduction in population) (46, 47). We estimated the distribution of the aggregate costs of dog vaccination campaigns by component (vaccines, syringe and needles, personnel, and overhead and other costs) based on the average cost distribution accrued during dog vaccination campaigns in Chad and Tanzania (41, 42, 44).

Daily vaccination capacity is likely to vary depending, among other factors, on vaccinator experience, dog density, and dog owner reception to vaccination (39, 50–53). A large-scale campaign in an African city was able to vaccinate approximately 100 dogs per person, per day (39). This figure is consistent with unpublished data collected by the authors of this analysis in urban, semi-urban, and rural areas of Haiti and was used for vaccinator capacity calculations. Other estimates for daily dog vaccination capacity (median number of dogs vaccinated per person per day) include ~9 in Mali (54), ~21 in Chad (51), ~50 in a different area of Chad (53), ~25 in Guatemala, and ~100 in Haiti (unpublished data collected by CDC). A much higher estimate was obtained in Malawi (39) where ~200 dogs were vaccinated per person per day in each static point station. We used a 50% increase to 150 dogs per day and a 100% increase to 200 dogs per person per day for our sensitivity analysis, because we were interested in showing the results from the best plausible scenarios of dog vaccination.

Last, we assumed a new technology for relatively inexpensive, effective management of dog populations would be developed; it would be capable of effecting a 40% decrease in the total dog population over a 5-year period and would cost US$8, similar to the current cost of sterilizing a female dog (46, 47, 49). We assumed that dog population management activities would require reasonable country infrastructure and would therefore begin during Phase II of the GDREP. The scenario required a one-time 70% sterilization of the female dog population to achieve a 40% population reduction and maintenance of this population through continued sterilization of 30% of female dogs (45, 46).

The results from our model are based on several critical assumptions, informed by previous literature, dog vaccination campaigns, and expert opinion. For transparency and ease of understanding, we provide a list of the main assumptions of our model and their rationale in Table 2.

**Table 2:** **Main assumptions that inform our estimates**.

Assumption	Rationale
70% of the dog population has to be vaccinated annually for 5–7 years to eliminate dog rabies	WHO recommendation; research suggests 70% threshold (7, 13, 14). Caveat: possibly varies by setting (55)
Dog vaccination coverage estimated by Hampson et al. (4) is reasonably accurate	Refereed review; provides country-specific estimates
Regional human-to-dog ratios estimated by Knobel et al. (6) are representative of countries in each region	We crossed-checked using human-to-dog ratio estimated from Davlin and VonVille (29) and found a ~3% aggregate difference
Countries where rabies has been eliminated from specific regions within the country (e.g., Brazil) still require national vaccination coverage	Our aim is not to explore detailed trends at the subnational level but to illustrate global trends
The time frames presented in the GDREP accurately reflect a country’s progression toward elimination	However, we recognize that between and within country capabilities and willingness to conduct dog rabies elimination campaigns using vaccines will, in reality, vary
All countries commit to dog rabies elimination at year 1 of GDREP and move through the phases as predicted	While this assumption is unlikely to reflect reality, this analysis and supplementary table can be used to forecast needs on a country-specific level
After 7 years of vaccination of 70% of dog population, we consider the country rabies free and do not longer estimate dog vaccination maintenance costs	Countries completing the GDREP will likely continue to fund rabies prevention programs and maintain some level of dog rabies vaccination. However, these activities are no longer for the purpose of elimination, rather they are for the purpose of preventing re-incursion of the virus. Therefore, these costs are not considered
Vaccination capacity: all public health veterinary work force would be willing/able to do dog vaccinations, and veterinary workers can move within countries at ease. We assume the workforce reported by OIE (40) is reasonably accurate	Larger cities may have an unequal distribution of vaccination capacity. This is not accounted for under the vaccinator capacity assessment

*WHO, World Health Organization; OIE, World Organization for Animal Health; GDREP, Global Dog Rabies Elimination Pathway*.

We conducted a robustness check on two key parameters. First, we assessed whether our estimate of dog population was reasonable, by calculating the global dog population based on human-to-dog ratios obtained from Davlin and VonVille (29). Second, we checked the robustness of the assumption that the current dog rabies vaccination coverage was an indicator of a country’s capability to conduct mass dog vaccination campaigns by comparing current dog vaccination rates to their UN-defined human development index (HDI). The HDI is an aggregate composite index of life expectancy at birth, mean of years of schooling, and gross national income per capita (38). The UN’s HDI is a method of quantifying the development of a country in a more robust manner than economic growth alone. We compared HDI to dog vaccination coverage among DECs, with mean, SD, and analysis of variance calculated to determine if significant differences were present. Development measures, such as HDI, have been used as benchmark for health system capabilities in other studies of disease burden (4, 56–59).

## Results

### Dog Populations

Our estimates show that there are approximately 687 million dogs globally (an average global human-to-dog ratio of 11:1), of which 536 million resided in the 122 DECs (78.1%) in 2015. Based on the WHO’s recommendation of 70% vaccination coverage during 7 years for elimination (7, 13, 14), a total of 375 million dogs would need to be immunized in DECs. An estimated ~130 million dog rabies vaccines were utilized in 2015, representing a gap of 246 million annual dog vaccinations to achieve the desired vaccination goal of 70% (country-specific estimates are shown in the Supplementary Material) (4). We compared these global estimates of dog population with estimates based on Davlin and VonVille’s (29) human-to-dog ratios. While differences were noted between individual country estimates, estimates of the global mean were within 2.5% difference, not statistically different (paired *t*-test, *p*-value = 0.96).

### Human Development Index

Seventy countries were determined to be dog rabies virus free; 122 were categorized as DECs. Countries free of dog rabies (*n* = 70) had a significantly higher mean HDI score of 0.78 compared to 0.60 for DECs (*p* < 0.05). Five mean HDI scores were calculated in accordance with the 13-year vaccination program phases and are displayed in Table 3. Only 5 DECs were estimated to be vaccinating more than 70% of dogs as of 2015 (Phase III); 56 (46%) were vaccinating less than 18% (Phase I). The remaining 61 DECs were defined as Phase II. The mean HDI score for DECs in Phase I was significantly lower than DECs in Phases II and III (mean 0.46 vs 0.71, *p* = 0.004). We interpreted this to suggest that significant structural improvements would be needed in these countries before sustained higher dog vaccination levels could be achieved, which validated our assumption that current dog vaccination coverage is a reasonable indicator of a country’s capabilities to conduct mass dog vaccination campaigns (specific details are shown in the Supplementary Material).

**Table 3:** **Association between Human Development Index (HDI) and dog rabies vaccine coverage and elimination**.

	Dog rabies free	Dog rabies endemic	Current dog vaccination coverage, endemic countries				
			>70%	53–69%	35–53%	18–35%	<18%
Countries	70	122	5	9	24	28	56
Total HDI score^a^	54.30	73.04	3.49	6.38	18.18	18.91	26.09
HDI range	0.43–0.95	0.28–0.89	0.63–0.77	0.57–0.89	0.45–0.88	0.43–0.85	0.28–0.77
Mean HDI score	0.78	0.60	0.70	0.71	0.76	0.68	0.47
95% CI	0.76–0.80	0.58–0.62	0.57–0.83	0.63–0.79	0.71–0.81	0.64–0.72	0.44–0.50
SD	0.13	0.16	0.05	0.10	0.06	0.12	0.12

*Test of significance; two-tailed t-test: p < 0.05; ANOVA: p = < 0.05*.

*CI, confidence interval*.

*^a^HDI is a composite measure of health, education, and income used by the United Nations Development Program to rank countries based on their human development (38)*.

### Dog Vaccines Required

**Figure 2:**
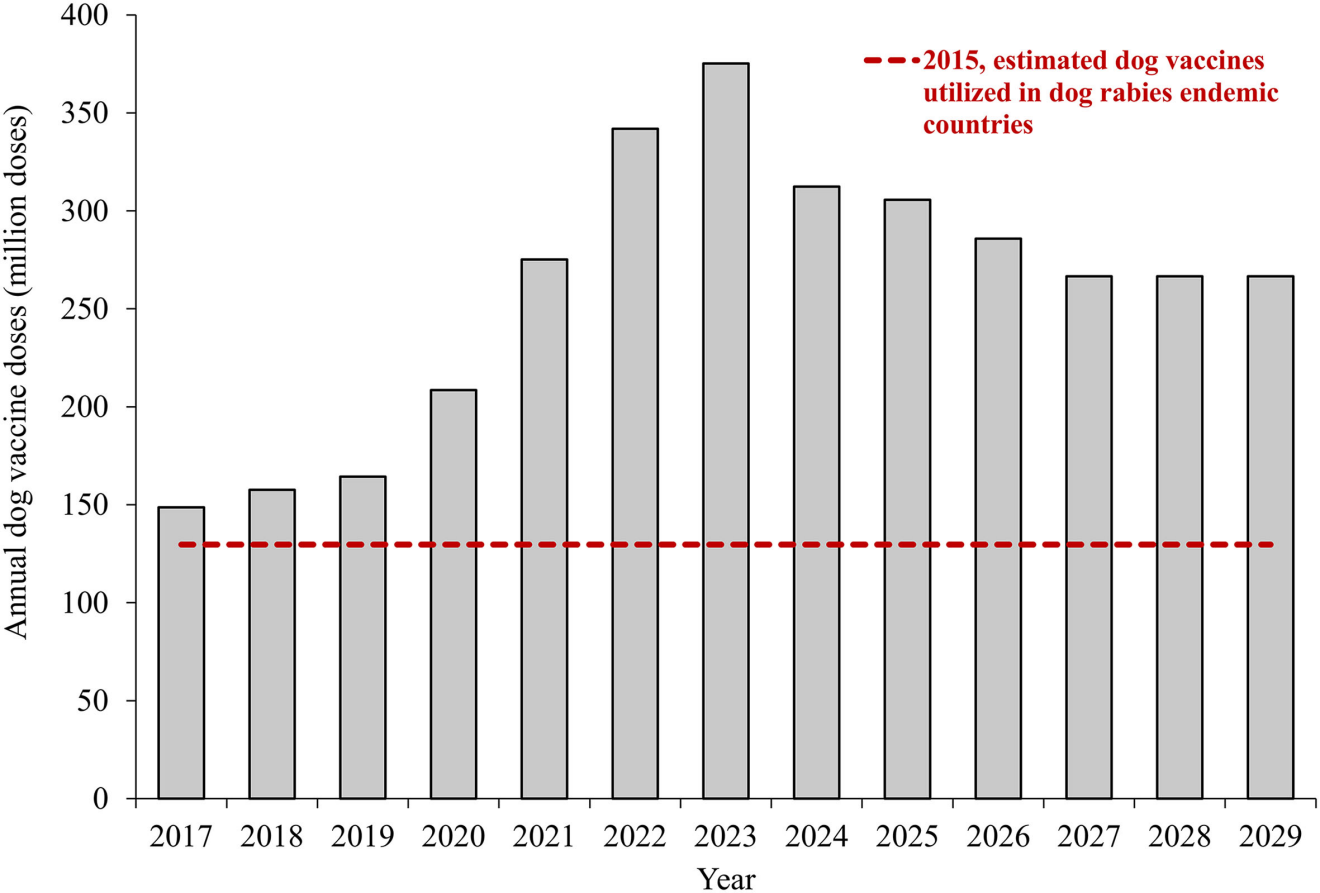
**Annual dog vaccinations required to achieve rabies elimination goal by 2030**. Notes: the estimates show aggregate values of canine vaccination requirements for countries, assuming that all countries begin working toward rabies elimination in the first year. The estimates are based on current human and dog population and vaccine availability.

Figure 2 shows the number of dog vaccinations required annually for the 13-year dog rabies elimination program. The line shows the number of dog vaccinations estimated to have occurred in 2015 as a reference (*n* ≈ 130 million). The results suggest that, if the current scenario does not change, by the second year of the GDREP, we would require additional production of at least 30 million doses. The largest gap in dog rabies vaccines is predicted to occur in year 7 of the program, with a potential gap of 245 million doses. However, this spike in dog vaccine needs is model dependent, as it corresponds to the point where we would expect the largest number of countries vaccinating at 70%, if all countries begin in year 1 and progress as expected. The cumulative dog rabies vaccine gap for the duration of the 13-year program is 7.5 billion doses, assuming vaccine production remains at the estimated 2015 level.

### Vaccination Costs

**Figure 3:**
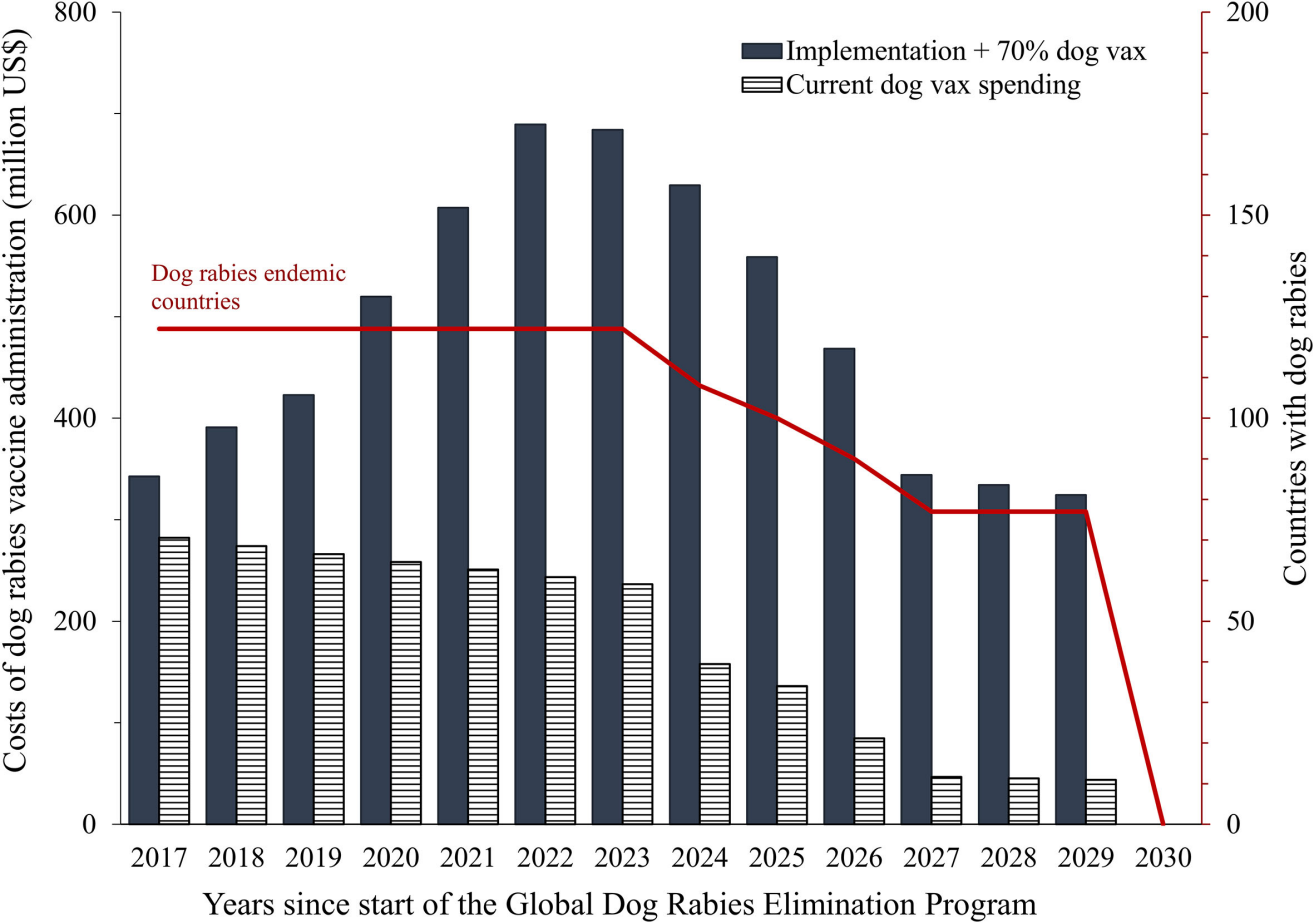
**Global annual costs of dog rabies vaccine administration to achieve dog rabies elimination in endemic countries, and number of countries with endemic rabies**. Notes: costs are in 2015 US dollars; we used a 3% discount rate (45).

Using an average cost per dog vaccinated of $2.18, based on estimates from vaccination campaigns in three developing countries (Table 1), we estimated a total present cost of $6,315 million to eliminate dog rabies in all DECs. Most DECs are currently vaccinating some proportion of their dog population, at an estimated value of $2,457 million over the course of the 13-year elimination program. Therefore, the additional present cost of dog vaccination to achieve elimination would be $3,858 million. An estimated $299 million is required to move all Phase I countries into Phase II; $1,386 million to move all Phase II countries to Phase III, and $4,631 million to move all countries through Phase III, elimination. Year 1 of the global elimination program is anticipated to have a funding gap of $60 million (Figure 3). The funding gap is anticipated to reach a peak in year 7 of the global campaign ($448 million).

**Figure 4:**
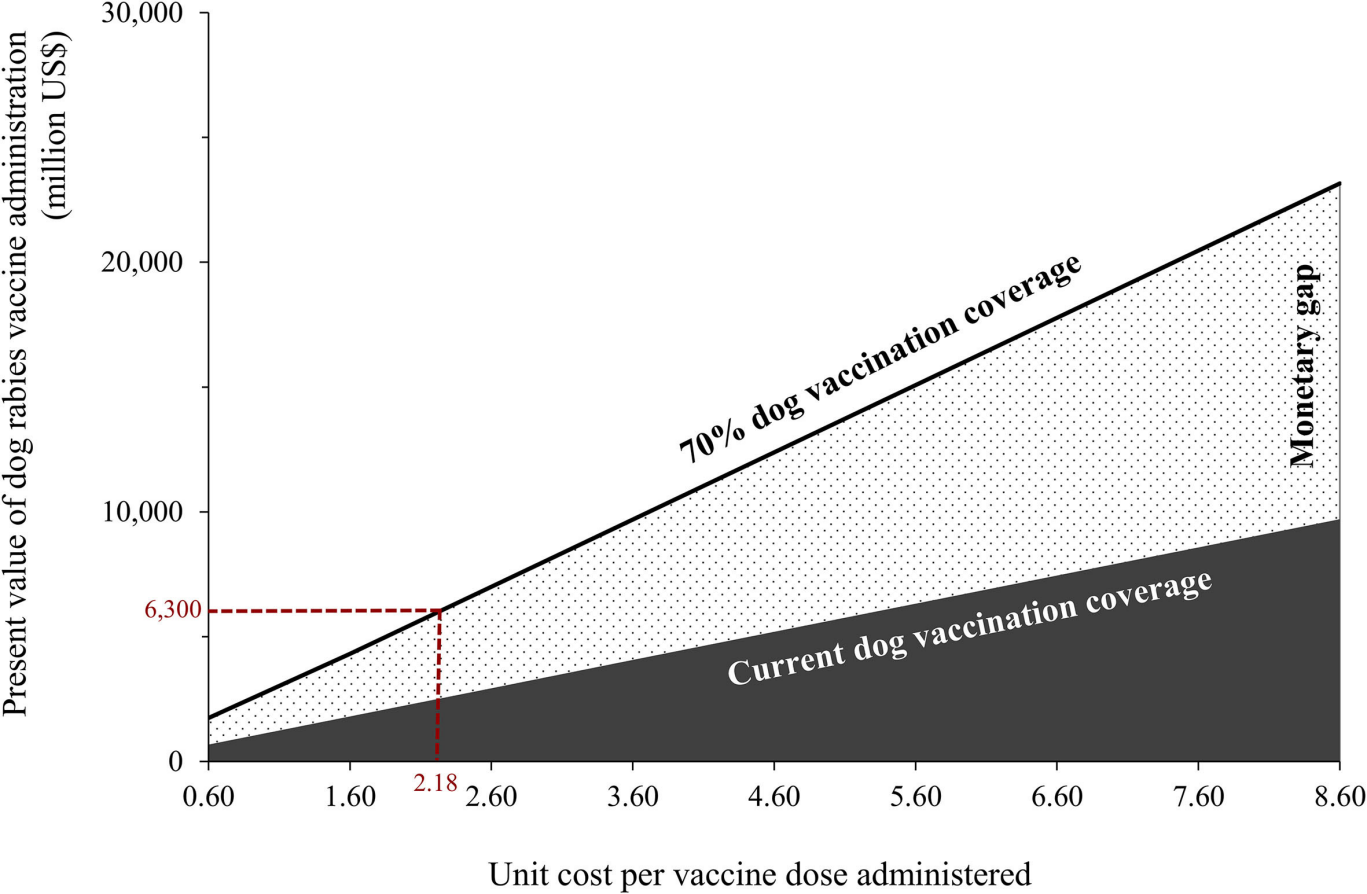
**Variability of the aggregate costs of GDREP based on the costs of administering rabies vaccines to 70% of the dog population, in rabies endemic countries, for a range of unit costs per dog vaccinated**. Notes: costs are in 2015 US dollars; we used a 3% discount rate (45). Unit costs per dog vaccinated were informed by previous economic studies of dog vaccination (6, 41–44, 49). GDREP denotes Global Dog Rabies Elimination Pathway.

The costs of vaccinating a dog vary substantially within and between countries (6, 41–44). Figure 4 shows the aggregate cost of eliminating dog rabies for a range of estimates of unit costs per dog vaccinated derived from previous studies. If the mean cost to vaccinate a dog was as high as $8.60, then the gap for global elimination could be $13.5 billion. If the cost to vaccinate a dog could be reduced to approximately $0.30, there would be no anticipated gap in funding to achieve global elimination. At $0.30 per dog vaccinated, this value is sevenfold lower than the current most likely cost ($2.18).

### Animal Health Workers’ Vaccination Capacity

Using data for animal health work force from OIE (40), we estimated each country’s dog vaccination capacity. We estimated that each worker would be able to vaccinate 100 dogs per day (39), and compared animal workforce requirements for a single 1-month vaccination campaign (25 work days) compared to a 3-month campaign.

**Figure 5:**
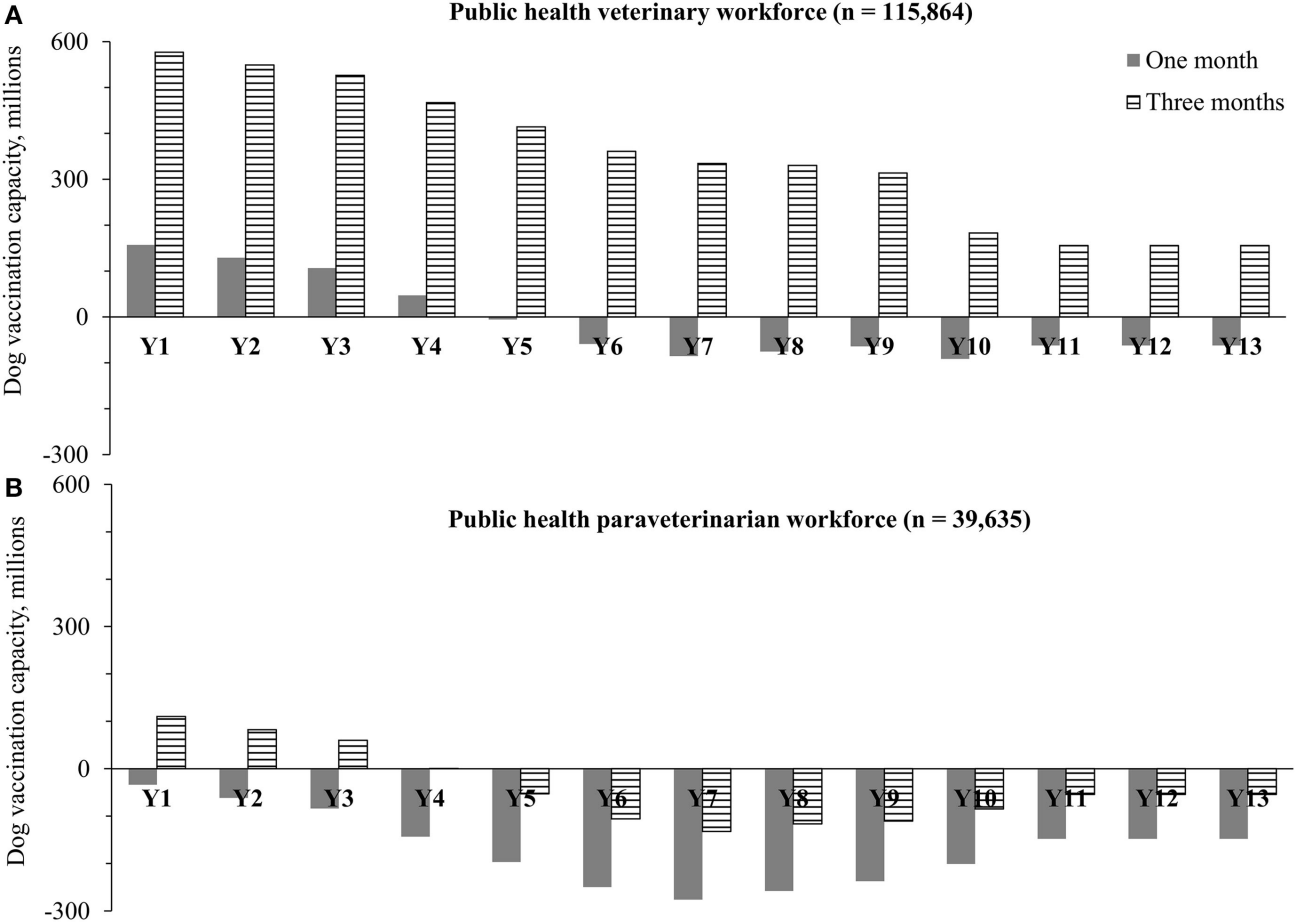
**Annual surplus or deficit of global dog vaccination capacity (total dogs potentially vaccinated by existing workforce) to achieve rabies elimination in dog rabies endemic countries based on (A) public health veterinary workforce and (B) public health para-veterinary workforce**. Notes: workforce data were obtained from the World Organization for Animal Health (40); the vaccination capacity was estimated for each country with dog endemic rabies and then aggregated at the global level. The estimates are based on a dog vaccination capacity of 100 dogs per worker per day (39).

Figure 5 shows the aggregate results for annual dog vaccination capacity (total dogs potentially vaccinated by existing workforce) for (A) all public health veterinary workforce (*n* = 115,864) and (B) all public health para-veterinarian workforce (*n* = 39,635). Under scenario (A), our estimates show that there will be a global shortage of dog vaccinators in year 5 of the GDREP, assuming 1-month vaccination programs are utilized. Dog vaccinator shortages would reach their peak in year 10 of GDREP, at 91 million dogs unable to be reached for vaccination. The vaccinator workforce may be adequate if they were to dedicate 3-months each year to dog rabies vaccination.

Under scenario (B), utilizing only the para-veterinarian workforce for dog vaccination, we would expect an immediate workforce shortage in year 1 under GDREP when utilizing a 1-month vaccination program. Dog vaccinator shortages under this method would peak in year 7, at 276 million dogs unvaccinated. If this para-veterinary workforce were to dedicate 3-months toward dog rabies vaccination, a shortage in workforce would still be expected in year 5 of the GDREP and peak in year 7 (expected shortage of 132 million dogs) (further details about country capacity by year are shown in the Supplementary Material).

### Sensitivity Analysis: Hypothetical Scenarios Based on Technological Improvements

The four scenarios based on hypothetical technological improvements to reasonably improve current dog vaccination practices included a 50% decrease in the price of dog rabies vaccine, a 50% and 100% increase in the daily vaccination capacity of a vaccinator and a 40% global reduction in the dog population (46, 47).

**Figure 6:**
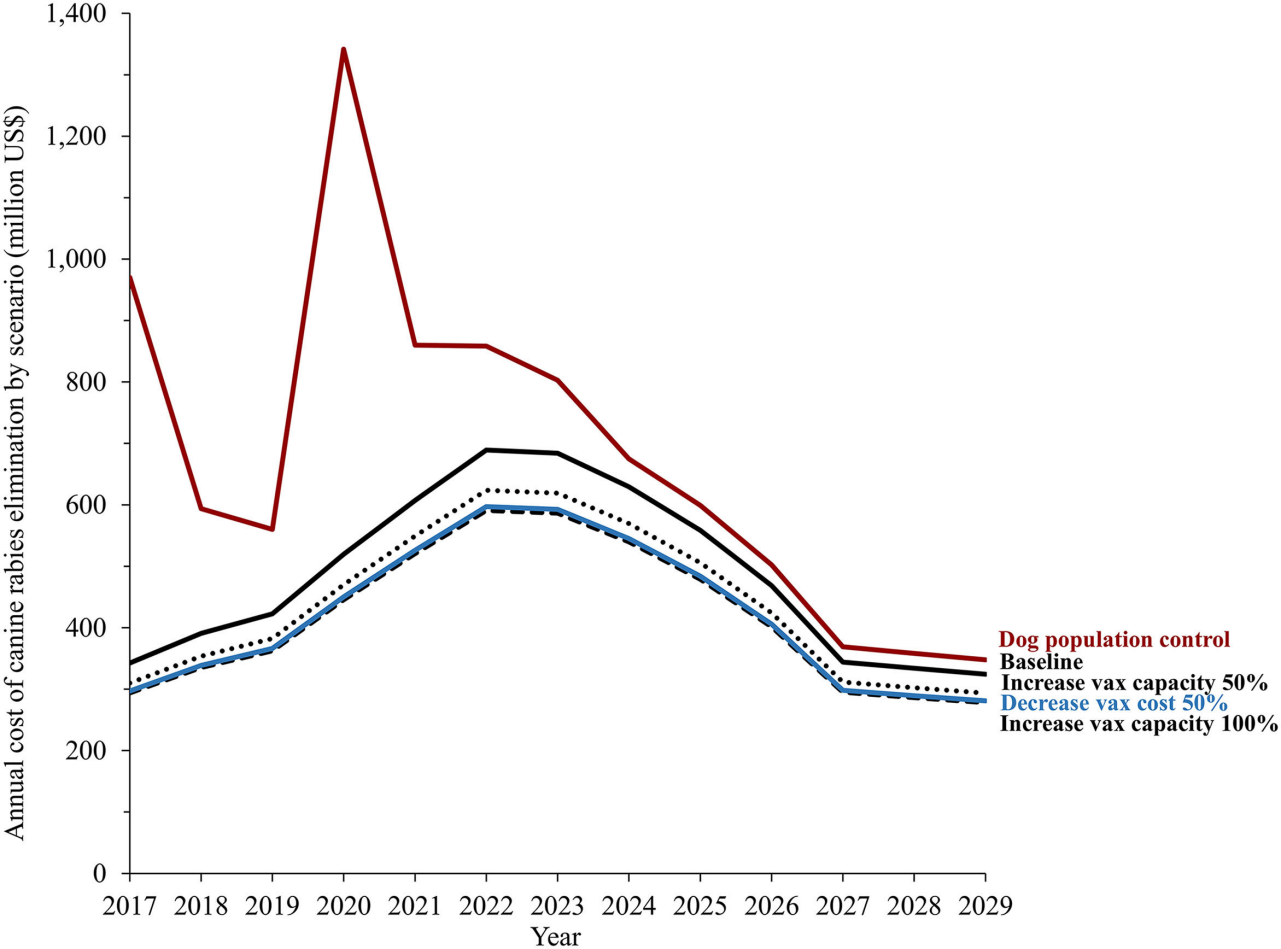
**Annual costs of dog rabies control under four hypothetical scenarios: decrease in the price of dog rabies vaccine, (including syringe and needle) increases in daily vaccination capacity of health workers, and effective dog population management and control**. Notes: the four hypothetical scenarios assuming that a new technology impacted the feasibility of dog vaccination, reasonably improving current practices. The estimates are based on current human and dog population and vaccine availability. The distribution of aggregate costs components was estimated based on previous literature (41, 42, 44).

Figure 6 shows the annual costs of dog rabies elimination in endemic countries under each of the four hypothetical scenarios. The results suggest that, based on the limited existing evidence of dog population management, massive sterilization campaigns with current technology are the costliest path toward global rabies elimination. The spike in aggregate costs around the fourth year corresponds to our assumption that countries currently lagging in dog vaccination would be able to conduct massive sterilization only once they have achieved the capabilities of implementing massive vaccination campaigns.

**Figure 7:**
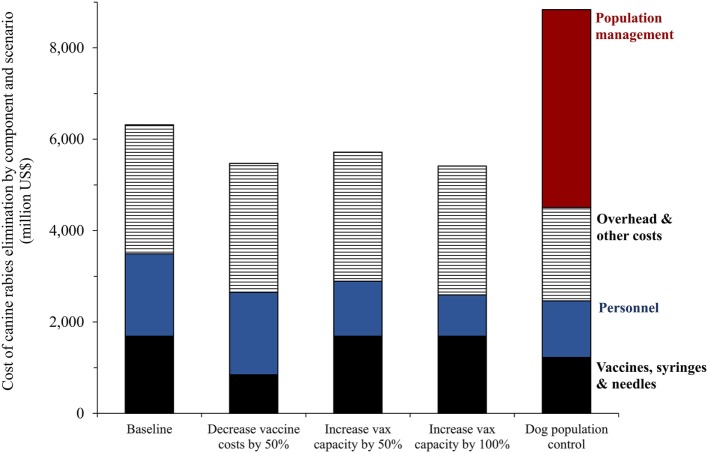
**Aggregate costs of dog rabies control (2017–2030) under four hypothetical scenarios: decrease in the price of dog rabies vaccine (including syringes and needles), increases in daily vaccination capacity of health workers, and effective dog population management and control**. Notes: the four hypothetical scenarios assuming that a new technology impacted the feasibility of dog vaccination, reasonably improving current practices. The distribution of aggregate costs components was estimated based on previous literature (41, 42, 44).

Figure 7 compares the total costs of each of these hypothetical scenarios. Reduction of the cost of the rabies vaccine (including syringe and needle) by 50% would equate to an overall 13% reduction in the global cost to eliminate dog rabies ($5,471 million). Increasing daily capacity to vaccinate dogs from 100 dogs per person to 150 dogs per person would result in an expected ~10% reduction in total program costs, and increasing the daily capacity to 200 dogs per person/day would yield a net cost reduction of ~14%. Based on an estimated cost for spay surgery of $8, a 40% reduction in the global dog population would result in a ~29% reduction in rabies vaccination program costs. However, the cost necessary to achieve global dog population reduction was estimated at $4,331 million, and thus, the overall program cost was ~40% higher than the current estimate for elimination based solely on dog vaccination with no population management. The costs per female dog sterilized would need to be reduced to less than half the price, about $3.50 per dog, to make the costs of dog population management plus dog vaccines comparable to that of only vaccinating dogs.

### Prioritization for Global Dog Rabies Elimination

**Figure 8:**
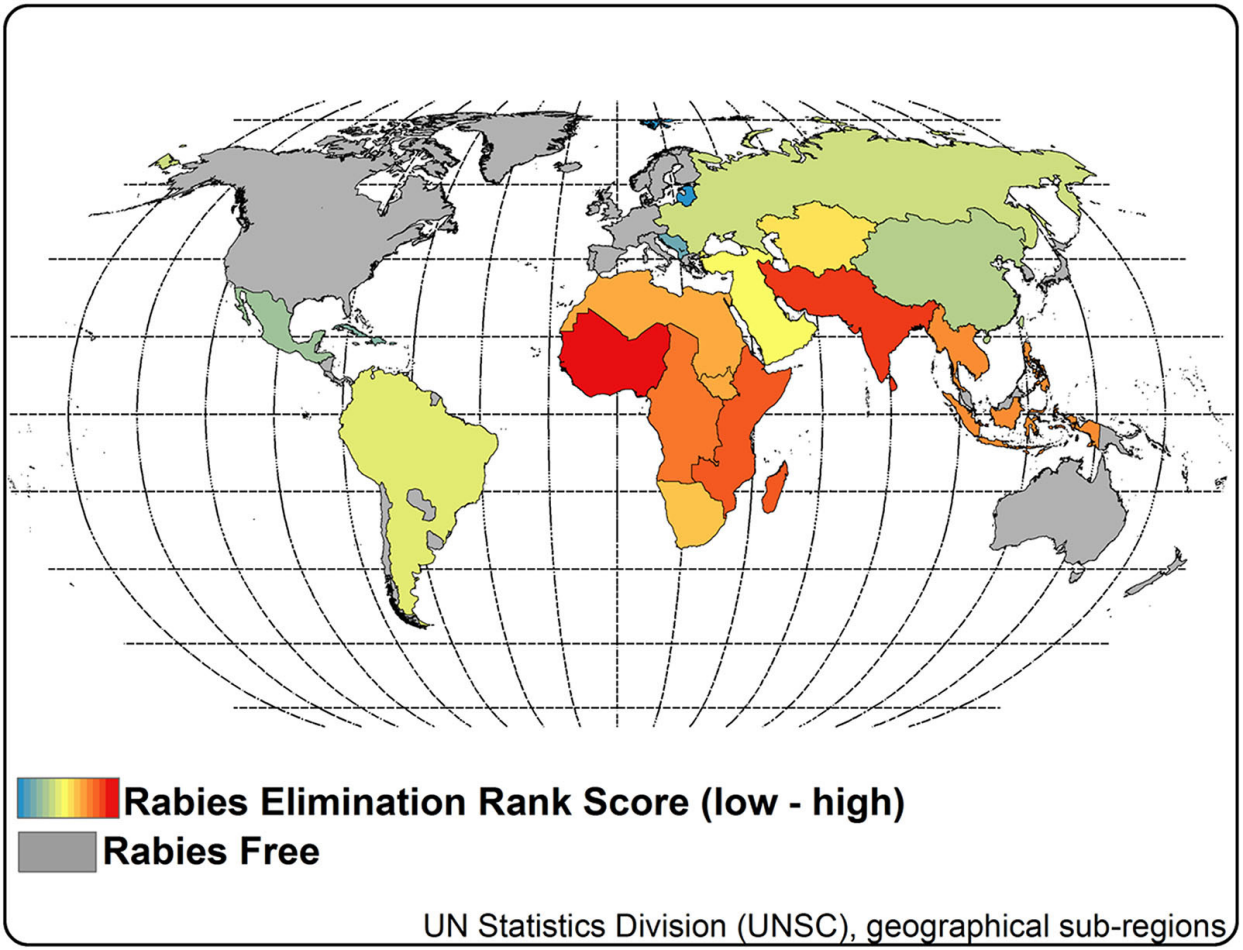
**Rabies elimination rank scores by rabies clusters**. Notes: elimination rank scores were estimated for each rabies cluster (4) based on six criteria: proportion of the cluster considered rabies free, funding gap for elimination, dog vaccination coverage for 2015 estimates, gap in vaccination workforce, average years to achieve elimination, and average human development index. Rank scores ranged from 16 to 84. A low rank score represents a theoretically quicker pathway toward elimination.

The UN define 22 clusters of countries. Of these clusters, six were free of dog rabies. Of the remaining 16 clusters in which dog rabies is currently endemic, our applied elimination scores ranged from 16 to 84 (Table 4). Clusters with the lowest elimination scores were primarily located in the Western Hemisphere and Europe (Figure 8). Countries with the highest scores were mainly located in Africa and Asia. The 16 ranked clusters were stratified into three groups. Group 1, consisting of seven countries, had a mean HDI of 0.77, compared to 0.65 and 0.47 for the second and third groups. Group 1, with the lowest elimination scores, had a mean elimination time of 5.4 years, compared to 10.1 and 11.7 years for Groups 1 and 2.

**Table 4:** **Elimination Rank Scores for the feasibility of dog rabies elimination**.

	United Nations Cluster	Elimination Rank Score^a^	Dog-variant endemic countries	Human Development Index (HDI) (mean)	Gap in funding (US$)	Current dog vaccination coverage (%)	Gap in vaccinator capacity^b^	Average years for elimination
Dog rabies free	Micronesia	0	0 of 5	0.65	$0	70.0	6	0.0
	Australia and New Zealand	0	0 of 2	0.92	$0	70.0	795	0.0
	Western Europe	0	0 of 8	0.90	$0	70.0	−4,435	0.0
	Polynesia	0	0 of 5	0.76	$0	70.0	8	0.0
	Melanesia	0	0 of 4	0.57	$0	70.0	−193	0.0
	Northern America	0	0 of 2	0.91	$0	76.1	−973	0.0

Group 1	Northern Europe	16	3 of 10	0.87	$444,538	68.6	−1,213	2.7
	Southern Europe	22	6 of 13	0.82	$2,395,923	64.8	−158	4.5
	Caribbean	28	3 of 13	0.72	$2,694,433	43.4	2,663	2.9
	Central America	31	4 of 8	0.68	$0	72.4	−6,689	5.0
	Eastern Asia	37	3 of 5	0.78	$35,848,426	36.8	21,315	7.8
	Eastern Europe	39	10 of 10	0.78	$18,956,245	46.2	36,090	8.9
	South America	40	9 of 13	0.73	$12,631,906	58.7	−4,947	5.9

Group 2	Western Asia	51	16 of 17	0.73	$25,414,328	31.1	3,704	9.5
	Central Asia	52	5 of 5	0.66	$5,394,654	28.9	5,165	10.4
	Southern Africa	56	5 of 5	0.57	$1,726,750	53.1	−1,239	12.0
	Northern Africa	60	6 of 6	0.63	$22,562,725	17.3	9,302	10.8
	South-Eastern Asia	63	7 of 11	0.64	$56,600,142	25.3	−9,725	7.6

Grou 3	Middle Africa	74	8 of 9	0.46	$20,372,954	0.6	−3,894	12.2
	Eastern Africa	75	14 of 17	0.44	$56,414,022	4.1	−3,606	11.4
	Southern Asia	78	8 of 9	0.55	$190,629,398	13.2	−19,724	10.8
	Western Africa	84	15 of 16	0.41	$42,702,854	6.7	−7,205	12.6

*^a^Country clusters were given a ranked score based on the following criteria: proportion of the cluster that was considered rabies free, HDI, estimated funding gap for elimination, current dog vaccination coverage, gap in vaccinator capacity, and expected years to achieve elimination. Each of the six criteria was ordered from most optimal to least optimal value and received the corresponding numerical score, we summed the country cluster rank scores to obtain a cumulative rabies elimination rank score. Lower scores represent UN Regions that appear to be more favorably situated to achieve the rabies elimination goal*.

*^b^Includes all vet professionals marked as “public health” and assumes they can vaccinate 100 dogs per day and they can work 25 days per year on rabies vaccination (1 month)*.

## Discussion

The goal set forth by WHO, OIE, FAO, and global experts for the elimination of dog-mediated human rabies deaths will undoubtedly be the impetus for numerous countries to improve their rabies control and elimination programs. Achieving this global target will take international coordination from governments, non-government entities, private industry, educational institutions, and many more partners. Establishing a framework that clearly describes the challenges that these partners will face is a critical first step in developing both regional and global strategies. There are several key approaches for how countries might achieve this goal; however, only mass vaccination of dogs has been effectively proven as a sustainable and cost-effective method. In this assessment, we have attempted to describe the scope of the resources that will be required to eliminate dog-mediated human rabies deaths through mass vaccination of dogs. In our attempt to conduct this global analysis, we made several critical assumptions to develop a model of an ideal scenario of dog rabies elimination. We focused on quantitative, measurable factors that affect a country’s ability to eliminate dog rabies, but there are many qualitative aspects of rabies control that would need careful consideration when assessing individual country prospects for elimination. The capacity to eliminate dog rabies varies by country, with unique challenges and opportunities that cannot be readily quantified or generalized. These include, but are not limited to, political support, economic support, political stability, veterinary capacity, dog ownership characteristics, legislation, and dog ecology. We acknowledge that there is variation between and within countries for the implementation and scaling-up of national dog vaccination campaigns. However, the purpose of this analysis was not to provide a detailed roadmap for countries to follow toward rabies elimination, but rather to provide evidence for regional and global leaders to continue to advocate for resources, be they monetary, human, or material, to support global rabies elimination efforts.

At first glance, the feasibility of global dog-mediated human rabies elimination through mass dog vaccination is sobering. Of the four main factors assessed in this analysis, three are likely to represent substantial barriers to this goal: country capabilities (measured by HDI), vaccine demand, and funding. We have predicted gaps in the availability of dog rabies vaccines to the order of hundreds of millions. The cost of global elimination is likely to be billions of dollars. In a recent WHO expert consultation (7), scaling-up of animal rabies vaccines was determined to be possible, but the degree of expansion was not reported. However, with more countries developing the technology to produce local animal rabies vaccines (e.g., Ethiopia, India, and China), and growing demand for dog vaccines, the regional supply could be expanded. Clearly, local capacity building, regional approaches, and joint attempts for funding mobilization would be critical components of elimination efforts.

As a neglected disease, rabies control will always be susceptible to priority shifting based on new agendas or more urgent public health threats. New tools like the Stepwise Approach toward Rabies Elimination and the One-Health Prioritization tool may help nations to identify if rabies is a priority and make necessary steps to develop sustainable elimination plans. Development of regional consortiums to support national elimination planning, coordinate efforts between countries, share surveillance data and technical assistance, and leverage regional resources may be critical in achieving global elimination. The Rabies Program Directors of the Americas have followed this model and have been a critical component of the successful elimination efforts in Latin America.

To assess the importance of a country’s overall human development and its relationship to public health programs, we adapted the HDI as an indicator of a country’s capabilities to conduct effective mass vaccination campaigns. While rabies elimination successes are not limited to high-income countries, dog rabies-free countries had a significantly higher HDI compared to countries that have not yet achieved elimination. This finding has two main implications for the interpretation of this study. First, it supports several assumptions used to create the GDREP, particularly the assumption that countries with lower vaccination coverage will probably require more time and monetary inputs before effective mass vaccination campaigns are realized. Second, this finding suggests that the resources available today may not be adequate for dog rabies elimination in the resource-poor countries that remain endemic. High levels of international support (ranging from monetary to technical assistance) have been provided in Tanzania, Chad, Malawi, and Haiti to achieve adequate dog vaccination coverage. As the global community prepares to provide support for dog rabies elimination, considerations for supporting national infrastructure should be considered.

World Organization for Animal Health vaccine bank mechanisms for lower cost procurement of vaccines is one of the most recent developments, which may facilitate vaccine acquisition and distribution. However, with the likely increase in dog vaccine demand, thorough analyses of global vaccine production capacity is needed. During rinderpest elimination AU-PANVAC was created to monitor quality of vaccine used in Africa (60). Similar approaches for vaccine capacity and quality monitoring should be adapted and implemented for GDREP.

This analysis shows that there may be an adequate veterinary workforce to vaccinate dogs to desired levels if this workforce can be utilized appropriately. This estimate has several potential limitations, including a lack of validation of the reported capacity in the OIE database and the assumption that the entire workforce would be able to dedicate time to vaccination of dogs. Each country is likely to address vaccination in a method that suits their specific capabilities. Vaccination of dogs has been carried out successfully by veterinarians, para-veterinarians, international organizations, and/or students, depending on the campaign design. Students were not considered a resource in this analysis, as they are unlikely to represent a reproducible and reliable resource in the majority of dog rabies endemic countries when considering mass vaccination on a national scale. While there may be scenarios in which there exists the capacity to vaccinate the required amount of dogs, diverting veterinary personnel to dog vaccination is likely to take them away from other disease control activities. Depending on the resources available, and the time commitment required, national vaccination programs would need to consider whether they have the current human capacity for rabies elimination or if more human capacity will need to be developed. The supplemental tables developed for this analysis could be used as a preliminary guide for national programs to determine what resources may be required.

While adequate resources for global rabies elimination appear to be lacking, it is not unrealistic to expect that new advances in control techniques and resources will be developed. We analyzed several hypothetical improvements to determine the potential impact they could have on rabies elimination. Dog rabies vaccines, including syringes and needles, account for approximately 27% of the cost to vaccinate a dog (41, 42, 44). While investments in cheaper vaccines are likely to have an impact toward the elimination goal, the costs of personnel and overhead and other costs represent a larger proportion of the cost to vaccinate a dog. There is vast documented variation in daily dog vaccination capacity (39, 50–53), which may be a relatively low-hanging fruit for cost reduction. By doubling a vaccinator’s daily efficiency from 100 to 200 dogs, the total cost to eliminate dog rabies dropped by over 14%. Current technologies such as oral rabies vaccination and applications that improve logistical coordination may be key to improving vaccinator efficiency.

Perhaps the most debatable hypothetical scenario considered in this analysis is the global reduction of the dog population. Currently, only surgical sterilization is used for large-scale operations, and the capacity to sterilize the required number of dogs does not exist globally. Therefore, this scenario assumes that new methods of population management will be developed. One such method is an injectable sterilizing agent, of which several candidates have appeared on the market in recent years. However, their use in mass sterilization has not yet been realized nor evaluated, and the cost for these injectable sterilizing agents is still prohibitive for most countries. Our analysis suggests that if an effective sterilizing agent was available at the current cost of $8 per sterilized female dog (including personnel and overhead), the estimated total cost for global elimination would still be substantially higher (~40%) than in the scenario with no population control. Only considering rabies control, to be comparable in terms of total costs an effective sterilizing agent would need to be produced at less than about $3.50 per dog (conditional on the assumptions in our model). Country-specific socio-cultural characteristics, legislation, and dog ownership, among other factors, would need close consideration in such scenario.

If we are to achieve the goal of dog-mediated human rabies elimination by 2030, we cannot wait for technological advances. It is also unlikely that, in the near-future, the global community will raise the total financial resources we predict are necessary to achieve this goal. Prioritization of countries or regional clusters for the finite available resources may be required. This study provides one possible method for considering resource prioritization through an elimination rank score. Three groupings of countries were identified, one group that appears to be nearing elimination, a group that is in the process of controlling dog rabies, and a group that appears to be at an early stage in their dog rabies control efforts. If prioritization of limited resources is a reality the rabies community must face, then international partners should address a global strategy where limited resources can be effectively distributed to begin making strategic regional progress toward the global target. Internationally sponsored vaccination programs of tens of thousands of dogs may benefit a community and assist with raising awareness or collect scientific data, but these small-scale vaccination efforts will not achieve elimination globally. If considering global elimination, there should be a discussion over whether resources are directed toward countries that are nearing elimination, to ensure they complete and thereby open up their resources and capacity to others sooner, or whether the focus should be directed toward countries with the highest rabies burden, where a larger reduction in human deaths from dog rabies would occur. In either case, if more resources are not allocated, and in a strategic manner, then global elimination of dog rabies by 2030 is unlikely to be achieved.

Achieving global dog rabies elimination will require unique regional and national strategies. Funding, vaccination methods, personnel, and technological advances will be utilized differently. Countries will progress at the pace set by their governments and with assistance by international supporters, not at the predicted pace of the 13-year elimination program utilized in this analysis. Natural disasters, human-made disasters, competing needs, political processes, economic stagnation, and other unpredictable events will undoubtedly derail rabies elimination efforts in some countries. But the information provided here can be used to discuss and advocate, in a quantifiable manner, the approximate resources that will be required, the technological advances that should be pursued, and the prioritization processes that may be necessary. We hope that these results help stimulate and inform a necessary discussion on global and regional strategic planning, resource mobilization, and continuous execution of rabies virus elimination.

## Author Contributions

All the authors have contributed substantially to the article and approve its contents. RW conceived the study. RW and EU collected and analyzed the data. RW, EU, JC, JB, and RF interpreted the data, wrote the article, and provided critical revision and interpretation of contents and implications.

## Disclaimer

The findings and conclusions in this article are those of the authors and do not necessarily represent the official position of the US Centers for Disease Control and Prevention.

## Conflict of Interest Statement

The authors declare that the research was conducted in the absence of any commercial or financial relationships that could be construed as a potential conflict of interest.

## Abbreviations

DEC, dog rabies endemic country; FAO, Food and Agriculture Organization; HDI, human development index; GDREP, global dog rabies elimination pathway; NGO, non-governmental organization; OIE, World Organization for Animal Health; PEP, post-exposure prophylaxis; RIG, rabies immunoglobulin; WHO, World Health Organization.
