# Supplementary material for: Elimination of Dog-Mediated Human Rabies Deaths by 2030: Needs Assessment and Alternatives for Progress Based on Dog Vaccination
Source: Front Vet Sci. 2017 Feb 10;4:9. doi: 10.3389/fvets.2017.00009 (PMC5300989; doi:10.3389/fvets.2017.00009)
Supplement: Supplementary file 1 [file data_sheet_1.pdf]

Supplementary Material

Elimination of Dog-Mediated Human Rabies Deaths by 2030: Needs Assessment and Alternatives for Progress

Ryan M. Wallace*, Eduardo A. Undurraga, Jesse D. Blanton, Julie Cleaton, and Richard Franka

Contents

1 Appendix A. Country-specific estimates for rabies elimination in 2030 2

1.1 Dog populations and vaccines required 2

1.2 Vaccination costs 9

1.3 Animal health workers vaccination capacity 14

2 Appendix 2. Human development index and dog rabies elimination 20

3 References for the supplementary material 21

* **Correspondence:** Ryan Wallace: euk5@cdc.gov

1 Appendix A. Country-specific estimates for rabies elimination in 2030

1.1 Dog populations and vaccines required

Table S1. Estimates of the dog population by country and dog vaccines required to reach goal of 70% of dog vaccination coverage, by country

Country	Country code	Cluster	WB Human Population 2015	Urban (%)	HDI	Urban Dog Pop.	Rural Dog Pop.	Total Dog Population	Vax dogs, endemic countries	Current Unvax Dogs	Vax to reach goal
Afghanistan	AFG	Eurasia	32,526,562	27	0.40	1,158,076	1,667,202	2,825,278	28,253	2,797,025	1,949,442
Albania	ALB	EasternEurope	2,889,167	57	0.74	255,167	189,320	444,487	207,576	236,912	103,566
Algeria	DZA	North Africa	39,666,519	71	0.70	1,323,346	1,569,132	2,892,479	685,517	2,206,961	1,339,218
Andorra	AND	EU	70,473	85	0.84	9,228	1,614	10,842	0	3,253	0
Angola	AGO	Congo Basin	25,021,974	44	0.49	519,914	1,891,864	2,411,778	12,059	2,399,719	1,676,186
Antigua and Barbuda	ATG	Caribbean	91,818	24	0.76	2,910	9,332	12,242	0	5,497	1,824
Argentina	ARG	Southern Cone	43,416,755	92	0.80	5,311,374	477,526	5,788,901	1,195,982	4,592,918	2,856,248
Armenia	ARM	EasternEurope	3,017,712	63	0.72	290,968	173,296	464,263	216,811	247,452	108,173
Australia	AUS	Oceania	23,781,169	89	0.93	2,835,445	175,897	3,011,342	0	903,403	0
Austria	AUT	EU	8,611,088	66	0.89	873,933	450,850	1,324,783	0	397,435	0
Azerbaijan	AZE	Eurasia	9,651,349	55	0.73	811,010	673,813	1,484,823	313,298	1,171,525	726,078
Bahamas	BHS	Caribbean	388,019	83	0.77	42,876	8,860	51,736	0	23,229	7,709
Bahrain	BHR	Middle East	1,377,237	89	0.81	163,019	10,811	173,830	32,159	141,671	89,522
Bangladesh	BGD	Asia 3	160,995,642	34	0.50	7,357,930	7,399,382	14,757,312	750,409	14,006,903	9,579,709
Barbados	BRB	Caribbean	284,215	31	0.79	11,928	25,968	37,895	0	17,015	5,646
Belarus	BLR	EasternEurope	9,513,000	77	0.76	1,122,051	341,487	1,463,538	683,472	780,066	341,004
Belgium	BEL	EU	11,285,721	98	0.89	1,699,074	37,191	1,736,265	0	520,879	0
Belize	BLZ	CA & Mex*	359,287	44	0.70	21,065	26,840	47,905	0	15,330	958
Benin	BEN	West Africa	10,879,829	44	0.43	225,551	824,074	1,049,625	8,397	1,041,228	726,340
Bhutan	BTN	Asia 3	774,830	39	0.52	39,923	33,245	73,168	7,317	65,852	43,901
Bolivia	BOL	Andean	10,724,705	69	0.66	979,695	450,266	1,429,961	1,110,567	319,393	0
Bosnia Herzegovina	BIH	EasternEurope	3,810,416	40	0.73	233,121	353,097	586,218	273,764	312,454	136,589

Country	Country code	Cluster	WB Human Population 2015	Urban (%)	HDI	Urban Dog Pop.	Rural Dog Pop.	Total Dog Population	Vax dogs, endemic countries	Current Unvax Dogs	Vax to reach goal
Botswana	BWA	SADC	2,262,485	57	0.63	61,305	130,111	191,416	957	190,459	133,034
Brazil	BRA	Brazil	207,847,528	86	0.72	23,746,442	3,966,562	27,713,004	19,080,403	8,632,601	318,700
Brunei Darussalam	BRN	Asia 1	423,188	77	0.84	43,561	6,747	50,308	0	15,092	0
Bulgaria	BGR	EasternEurope	7,177,991	74	0.77	816,612	287,694	1,104,306	515,711	588,595	257,303
Burkina Faso	BFA	West Africa	18,105,570	30	0.33	255,007	1,716,139	1,971,146	15,769	1,955,376	1,364,033
Burundi	BDI	Congo Basin	11,178,921	12	0.32	63,577	1,328,524	1,392,102	6,961	1,385,141	967,511
C_te d'Ivoire	CIV	West Africa	22,701,556	54	0.40	580,175	1,405,656	1,985,830	15,887	1,969,944	1,374,195
Cambodia	CMR	Asia 2	15,577,899	21	0.52	430,428	863,615	1,294,042	7,764	1,286,278	898,065
Cameroon	CAN	West Africa	23,344,179	54	0.48	598,811	1,439,106	2,037,917	16,303	2,021,613	1,410,238
Canada	CSS	NorthAmerica	35,851,774	82	0.91	3,911,572	868,665	4,780,237	0	1,434,071	0
Cape Verde	CPV	West Africa	520,502	66	0.57	16,088	24,248	40,336	0	40,014	27,913
Central African Rep.	CAF	Congo Basin	4,900,274	40	0.34	92,544	397,074	489,618	2,448	487,170	340,285
Chad	TCD	West Africa	14,037,472	22	0.33	148,791	1,470,691	1,619,481	12,956	1,606,525	1,120,681
Chile	CHL	Southern Cone	17,948,141	90	0.81	2,142,529	250,556	2,393,085	0	842,366	124,440
China	CHN	China	1,371,220,000	56	0.69	15,788,619	12,601,029	28,389,648	4,031,330	24,358,318	15,841,424
Colombia	COL	Andean	48,228,704	76	0.71	4,915,212	1,515,282	6,430,494	4,707,879	1,722,614	0
Comoros	COM	SADC	788,474	28	0.43	10,524	76,401	86,925	0	86,490	60,413
Congo	COG	Congo Basin	4,620,330	65	0.53	142,489	216,157	358,646	277	358,369	250,775
Cook Islands	COK	Oceania	18,027	100	0.91	2,404	0	2,404	0	721	0
Costa Rica	CRI	CA & Mex*	4,807,850	77	0.74	492,458	148,588	641,047	0	500,016	307,702
Croatia	HRV	EasternEurope	4,224,404	59	0.80	383,212	266,696	649,908	303,507	346,401	151,429
Cuba	CUB	Caribbean	11,389,562	77	0.78	1,170,452	348,156	1,518,608	981,703	536,905	81,323
Cyprus	CYP	EasternEurope	1,165,300	67	0.84	119,974	59,303	179,277	0	95,555	41,772
Czech Republic	CZE	EasternEurope	10,551,219	73	0.87	1,184,853	438,411	1,623,264	758,065	865,200	378,221
North Korea[a]	PRK	Asia 2	25,155,317	61	0.77	2,041,773	688,253	2,730,026	16,380	2,713,646	1,894,638
DRC[b]	COD	Congo Basin	77,266,814	42	0.29	1,548,762	6,004,467	7,553,229	37,766	7,515,463	5,249,494
Denmark	DNK	EU	5,676,002	88	0.90	765,614	107,617	873,231	0	261,969	0
Djibouti	DJI	North Africa	887,861	77	0.43	32,391	27,184	59,576	60	59,516	41,643
Dominica	DMA	Caribbean	72,680	70	0.72	6,739	2,952	9,691	0	4,351	1,444
Dominican Republic	DOM	Caribbean	10,528,391	79	0.69	1,108,710	295,076	1,403,785	374,758	1,029,027	607,891
Ecuador	ECU	Andean	16,144,363	64	0.72	1,372,099	780,483	2,152,582	1,689,296	463,285	0

Country	Country code	Cluster	WB Human Population 2015	Urban (%)	HDI	Urban Dog Pop.	Rural Dog Pop.	Total Dog Population	Vax dogs, endemic countries	Current Unvax Dogs	Vax to reach goal
Egypt	EGY	North Africa	91,508,084	43	0.64	1,861,887	7,031,902	8,893,789	2,107,828	6,785,961	4,117,824
El Salvador	SLV	CA & Mex*	6,126,583	67	0.67	545,070	271,808	816,878	536,689	280,189	35,126
Equatorial Guinea	GNQ	Congo Basin	845,060	40	0.54	15,914	68,606	84,520	423	84,098	58,742
Eritrea	ERI	North Africa	5,222,000	38	0.35	92,863	439,636	532,499	532	531,966	372,217
Estonia	EST	EasternEurope	1,311,998	68	0.84	136,323	65,523	201,846	94,262	107,584	47,030
Ethiopia	ETH	SADC	99,390,750	19	0.36	912,895	10,815,863	11,728,757	58,644	11,670,113	8,151,486
Fiji	FJI	Oceania	892,145	54	0.69	63,911	28,868	92,779	0	27,834	0
Finland	FIN	EU	5,482,013	84	0.88	710,309	133,078	843,387	0	253,016	0
France	FRA	EU	66,808,385	80	0.88	8,173,235	2,104,978	10,278,213	0	3,083,464	0
French Guiana	GUF	Americas	276,000	84	0.816	31,059	5,741	36,800	0	11,040	0
Gabon	GAB	Congo Basin	1,725,292	87	0.67	70,929	29,945	100,875	794	100,081	69,819
Gambia	GMB	West Africa	1,990,924	60	0.42	56,001	108,608	164,609	1,317	163,292	113,909
Georgia	GEO	Eurasia	3,679,000	54	0.73	303,608	262,392	566,000	119,426	446,574	276,774
Germany	DEU	EU	81,413,145	75	0.91	9,431,525	3,093,574	12,525,099	0	3,757,530	0
Ghana	GHA	West Africa	27,409,893	54	0.54	698,720	1,702,303	2,401,022	19,208	2,381,814	1,661,507
Greece	GRC	EasternEurope	10,823,732	78	0.86	1,298,964	366,225	1,665,190	0	887,546	387,989
Grenada	GRD	Caribbean	106,825	36	0.75	5,069	9,174	14,243	0	6,395	2,122
Guatemala	GTM	CA & Mex*	16,342,897	52	0.57	1,123,759	1,055,294	2,179,053	1,372,803	806,250	152,534
Guinea	GIN	Congo Basin	12,608,590	37	0.34	221,013	1,070,691	1,291,704	6,459	1,285,245	897,734
Guinea-Bissau	GNB	West Africa	1,844,325	49	0.35	42,917	126,281	169,199	1,354	167,845	117,085
Guyana	GUY	CA & Mex*	767,085	29	0.63	29,203	73,075	102,278	69,549	32,729	2,046
Haiti	HTI	Caribbean	10,711,067	59	0.45	837,534	590,608	1,428,142	448,572	979,570	551,127
Honduras	HND	CA & Mex*	8,075,060	55	0.63	589,264	487,411	1,076,675	887,998	188,676	0
Hungary	HUN	EasternEurope	9,844,686	71	0.82	1,078,781	435,786	1,514,567	707,303	807,264	352,894
Iceland	ISL	EU	330,823	94	0.90	47,912	2,984	50,896	0	15,269	0
India	IND	India	1,311,050,527	33	0.55	57,243,962	61,658,798	118,902,760	17,835,414	101,067,346	65,396,518
Indonesia	IDN	Indonesia	257,563,815	54	0.62	18,455,993	8,331,739	26,787,732	6,375,480	20,412,252	12,375,932
Iran[c]	IRN	Middle East	79,109,272	73	0.71	7,739,524	1,472,926	9,212,450	1,674,991	7,537,459	4,773,724
Iraq	IRQ	Middle East	36,423,395	69	0.57	3,373,826	777,601	4,151,428	768,014	3,383,413	2,137,985
Ireland	IRL	EU	4,640,703	63	0.91	451,512	262,442	713,954	0	214,186	0
Israel	ISR	Middle East	8,380,400	92	0.89	1,029,560	46,063	1,075,623	679,567	396,056	73,369

Country	Country code	Cluster	WB Human Population 2015	Urban (%)	HDI	Urban Dog Pop.	Rural Dog Pop.	Total Dog Population	Vax dogs, endemic countries	Current Unvax Dogs	Vax to reach goal
Italy	ITA	EU	60,802,085	69	0.87	6,451,008	2,903,159	9,354,167	0	2,806,250	0
Jamaica	JAM	Caribbean	2,725,941	55	0.73	199,132	164,327	363,459	0	163,193	54,155
Japan	JPN	Asia 1	126,958,472	93	0.90	15,827,151	577,262	16,404,412	0	4,921,324	0
Jordan	JOR	Middle East	7,594,547	84	0.70	847,339	86,679	934,018	172,793	761,224	481,019
Kazakhstan	KAZ	Eurasia	17,544,126	53	0.75	1,245,563	573,595	1,819,158	383,842	1,435,315	889,568
Kenya	KEN	SADC	46,050,302	26	0.51	556,557	4,628,553	5,185,110	25,926	5,159,185	3,603,652
Kiribati	KIR	Oceania	112,423	44	0.62	6,641	4,379	11,020	0	3,306	0
Kuwait	KWT	Middle East	3,892,115	98	0.76	510,344	4,513	514,857	95,249	419,609	265,151
Kyrgyzstan	KGZ	Eurasia	5,957,000	36	0.62	283,609	267,828	551,436	116,353	435,083	269,652
Lao[d]	LAO	Asia 2	6,802,023	39	0.52	350,204	291,992	642,197	8,927	633,270	440,611
Latvia	LVA	EasternEurope	1,978,440	67	0.81	205,094	99,281	304,375	142,143	162,232	70,919
Lebanon	LBN	Middle East	5,850,743	88	0.74	684,865	49,948	734,813	135,940	598,872	378,429
Lesotho	LSO	SADC	2,135,022	27	0.45	27,506	209,717	237,222	1,186	236,036	164,870
Liberia	LBR	West Africa	4,503,438	50	0.33	105,578	306,106	411,684	3,293	408,391	284,885
Libya	LBY	North Africa	6,278,438	79	0.76	232,640	181,956	414,596	98,259	316,337	191,958
Lithuania	LTU	EasternEurope	2,910,199	67	0.81	297,772	149,951	447,723	209,087	238,636	104,319
Luxembourg	LUX	EU	569,676	90	0.87	79,018	8,624	87,642	0	26,293	0
Madagascar	MDG	SADC	24,235,390	35	0.48	401,313	2,125,345	2,526,658	12,633	2,514,025	1,756,028
Malawi	MWI	SADC	17,215,232	16	0.40	132,135	1,947,834	2,079,969	10,400	2,069,569	1,445,578
Malaysia	MYS	Asia 1	30,331,007	75	0.76	3,021,171	536,519	3,557,690	0	1,067,307	0
Maldives	MDV	Asia 1	409,163	46	0.66	24,842	15,584	40,426	0	12,128	0
Mali	MLI	West Africa	17,599,694	40	0.36	331,372	1,429,000	1,760,372	14,083	1,746,289	1,218,178
Malta	MLT	EasternEurope	431,333	95	0.83	63,311	3,048	66,359	0	35,369	15,462
Marshall Islands	MHL	Oceania	52,993	73	0.56	5,136	1,012	6,148	0	1,844	0
Mauritania	MRT	West Africa	4,067,564	60	0.45	114,849	220,643	335,493	2,684	332,809	232,161
Mauritius	MUS	SADC	1,262,605	40	0.73	23,627	102,935	126,562	0	125,929	87,960
Mexico	MEX	CA & Mex*	127,017,224	79	0.77	13,420,809	3,514,821	16,935,630	12,955,757	3,979,873	0
Micronesia[e]	FSM	Oceania	104,460	22	0.64	3,123	5,667	8,790	0	2,637	0
Moldova	MDA	EasternEurope	3,554,150	45	0.65	246,029	300,763	546,792	255,352	291,440	127,403
Monaco	MCO	EU	37,731	100	0.95	5,805	0	5,805	0	1,741	0
Mongolia	MNG	Eurasia	2,959,134	72	0.65	284,235	57,858	342,093	51,314	290,779	188,151

Country	Country code	Cluster	WB Human Population 2015	Urban (%)	HDI	Urban Dog Pop.	Rural Dog Pop.	Total Dog Population	Vax dogs, endemic countries	Current Unvax Dogs	Vax to reach goal
Morocco	MAR	North Africa	34,377,511	60	0.58	976,111	1,849,185	2,825,295	395,864	2,429,431	1,581,842
Mozambique	MOZ	SADC	27,977,863	32	0.32	425,132	2,562,848	2,987,979	298,798	2,689,181	1,792,788
Myanmar	MMR	Asia 2	53,897,154	34	0.48	2,450,380	2,483,867	4,934,248	29,605	4,904,642	3,424,368
Namibia	NAM	SADC	2,458,830	47	0.63	54,117	177,235	231,353	1,157	230,196	160,790
Nauru	NRU	Oceania	10,222	100	0.66	1,363	0	1,363	0	409	0
Nepal	NPL	Asia 3	28,513,700	19	0.46	707,710	1,622,788	2,330,498	3,962	2,326,537	1,627,387
Netherlands	NLD	EU	16,936,520	90	0.91	2,357,980	247,638	2,605,618	0	781,686	0
New Zealand	NZL	Oceania	4,595,700	86	0.91	528,714	44,080	572,794	0	171,838	0
Nicaragua	NIC	CA & Mex*	6,082,032	59	0.59	476,661	334,277	810,938	0	102,989	0
Niger	NER	West Africa	19,899,120	19	0.30	175,826	2,185,354	2,361,179	18,889	2,342,290	1,633,936
Nigeria	NGA	West Africa	182,201,962	48	0.46	4,106,076	12,858,534	16,964,610	2,085,290	14,879,320	9,789,937
Niue	NIU	Oceania	1,591	100	0.91	212	0	212	0	64	0
Norway	NOR	EU	5,195,921	80	0.94	643,279	156,093	799,372	0	239,812	0
Oman	OMN	Middle East	4,490,541	78	0.71	464,867	70,213	535,079	80,262	454,817	294,294
Pakistan	PAK	Asia 3	188,924,874	39	0.50	9,763,134	8,091,005	17,854,139	907,883	16,946,256	11,590,014
Palau	PLW	Oceania	21,291	87	0.78	2,472	192	2,664	0	799	0
Panama	PAN	CA & Mex*	3,929,141	67	0.77	348,866	175,020	523,885	0	488,960	331,794
Papua New Guinea	PNG	Oceania	7,619,321	13	0.47	132,119	463,526	595,645	0	178,694	0
Paraguay	PRY	Southern Cone	6,639,123	60	0.67	528,173	357,043	885,216	0	144,290	0
Peru	PER	Andean	31,376,670	79	0.73	3,288,652	894,904	4,183,556	1,369,595	2,813,961	1,558,895
Philippines	PHL	Asia 4	100,699,395	44	0.64	5,957,779	3,917,206	9,874,985	987,499	8,887,487	5,924,991
Poland	POL	EasternEurope	37,999,494	61	0.81	3,539,156	2,306,920	5,846,076	3,647,951	2,198,125	444,302
Portugal	PRT	EU	10,348,648	63	0.81	1,010,474	581,626	1,592,100	0	477,630	0
Qatar	QAT	Middle East	2,235,355	99	0.83	295,794	1,182	296,976	54,941	242,035	152,943
Republic of Korea	KOR	Asia 1	50,617,045	82	0.90	5,566,120	620,360	6,186,480	0	1,855,944	0
Romania	ROU	EasternEurope	19,832,389	55	0.78	1,664,822	1,386,315	3,051,137	1,424,881	1,626,256	710,915
Russian Federation	RUS	Eurasia	144,096,812	74	0.76	14,219,089	2,619,136	16,838,225	6,735,290	10,102,935	5,051,468
Rwanda	RWA	Congo Basin	11,609,666	29	0.43	157,776	1,116,866	1,274,642	331,407	943,235	560,842
Saint Kitts Nevis	KNA	Caribbean	55,572	32	0.74	2,375	5,035	7,410	0	3,327	1,104
Saint Lucia	LCA	Caribbean	184,999	19	0.72	4,564	20,102	24,667	0	11,075	3,675
St Vincent & Gren.	VCT	Caribbean	109,462	51	0.72	7,378	7,217	14,595	0	6,553	2,175

Country	Country code	Cluster	WB Human Population 2015	Urban (%)	HDI	Urban Dog Pop.	Rural Dog Pop.	Total Dog Population	Vax dogs, endemic countries	Current Unvax Dogs	Vax to reach goal
Samoa	WSM	Oceania	193,228	19	0.69	4,920	10,932	15,852	0	4,756	0
San Marino	SMR	EU	31,781	94	0.94	4,605	284	4,889	0	1,467	0
Sao Tome &Principe	STP	West Africa	190,344	65	0.51	5,844	8,979	14,823	0	14,705	10,258
Saudi Arabia	SAU	Middle East	31,540,372	83	0.77	3,495,935	372,088	3,868,023	715,584	3,152,439	1,992,032
Senegal	SEN	West Africa	15,129,273	44	0.46	312,013	1,150,622	1,462,635	11,701	1,450,934	1,012,143
Serbia and Montenegro	SRB	EasternEurope	7,098,247	56	0.77	606,660	485,378	1,092,038	509,982	582,056	254,445
Seychelles	SYC	SADC	92,900	54	0.77	2,361	5,789	8,150	0	8,110	5,665
Sierra Leone	SLE	West Africa	6,453,184	40	0.34	121,582	523,737	645,319	5,163	640,156	446,560
Singapore	SGP	Asia 1	5,535,002	100	0.87	738,000	0	738,000	0	221,400	0
Slovakia	SVK	EasternEurope	5,424,050	54	0.83	447,259	387,210	834,469	389,697	444,772	194,431
Slovenia	SVN	EasternEurope	2,063,768	50	0.88	157,640	159,863	317,503	148,274	169,229	73,978
Solomon Islands	SLB	Oceania	583,591	22	0.51	17,375	31,698	49,073	0	14,722	0
Somalia	SOM	North Africa	10,787,104	40	0.28	201,246	881,175	1,082,421	1,082	1,081,338	756,612
South Africa	ZAF	SADC	54,956,920	65	0.62	1,679,841	2,614,093	4,293,934	2,705,178	1,588,756	300,575
Spain	ESP	EU	46,418,269	80	0.88	5,682,953	1,458,319	7,141,272	0	2,142,382	0
Sri Lanka	LKA	Asia 4	20,966,000	18	0.69	513,136	1,197,027	1,710,163	843,110	867,052	354,004
Sudan	SDN	North Africa	52,574,694	34	0.41	838,368	4,702,877	5,541,245	11,082	5,530,163	3,867,789
Suriname	SUR	CA & Mex*	542,975	66	0.68	47,813	24,584	72,397	49,230	23,167	1,448
Swaziland	SWZ	SADC	1,286,970	21	0.52	12,935	136,857	149,792	749	149,043	104,106
Sweden	SWE	EU	9,798,871	86	0.90	1,293,677	213,842	1,507,519	0	452,256	0
Switzerland	CHE	EU	8,286,976	74	0.90	942,318	332,601	1,274,919	0	382,476	0
Syrian Arab Republic	SYR	Middle East	18,502,413	58	0.63	1,422,367	547,878	1,970,245	364,495	1,605,750	1,014,676
Tajikistan	TJK	Eurasia	8,481,855	27	0.61	302,881	434,283	737,164	108,435	628,729	407,580
Thailand	THA	Asia 4	67,959,359	50	0.68	4,564,513	2,358,427	6,922,940	3,426,855	3,496,085	1,419,203
Macedonia[g]	MKD	EasternEurope	2,078,453	76	0.73	242,699	77,063	319,762	149,329	170,433	74,505
Timor-Leste	TLS	Indonesia	1,245,015	33	0.50	54,399	58,533	112,932	0	86,054	52,175
Togo	TGO	West Africa	7,304,578	40	0.44	137,698	592,618	730,317	60,312	670,004	450,909
Tonga	TON	Oceania	106,170	24	0.70	3,357	5,664	9,021	0	2,706	0
Trinidad and Tobago	TTO	Caribbean	1,360,088	8	0.76	15,315	166,030	181,345	0	81,424	27,020
Tunisia	TUN	North Africa	11,107,800	67	0.70	350,221	497,720	847,940	410,827	437,113	182,731

Country	Country code	Cluster	WB Human Population 2015	Urban (%)	HDI	Urban Dog Pop.	Rural Dog Pop.	Total Dog Population	Vax dogs, endemic countries	Current Unvax Dogs	Vax to reach goal
Turkey	TUR	EasternEurope	78,665,830	73	0.70	8,882,824	3,219,611	12,102,435	5,651,837	6,450,598	2,819,867
Turkmenistan	TKM	Eurasia	5,373,502	50	0.69	358,499	187,746	546,244	115,258	430,987	267,113
Tuvalu	TUV	Oceania	9,916	60	0.58	790	279	1,069	0	321	0
Uganda	UGA	SADC	39,032,383	16	0.45	296,444	4,425,376	4,721,819	472,182	4,249,637	2,833,091
Ukraine	UKR	EasternEurope	45,198,200	70	0.73	4,846,290	2,107,279	6,953,569	3,247,317	3,706,252	1,620,182
U. Arab Emirates	ARE	Middle East	9,156,963	86	0.85	1,044,394	92,588	1,136,982	210,342	926,640	585,546
United Kingdom	GBR	EU	65,138,232	83	0.86	8,276,764	1,744,502	10,021,266	0	3,006,380	0
Tanzania[h]	TZA	SADC	53,470,420	32	0.47	797,214	4,941,823	5,739,037	28,695	5,710,341	3,988,630
USA[i]	USA	NorthAmerica	321,418,820	82	0.91	34,977,653	7,878,190	42,855,843	0	9,974,122	0
Uruguay	URY	Southern Cone	3,431,555	95	0.78	436,087	21,454	457,541	0	161,054	23,792
Uzbekistan	UZB	Eurasia	31,299,500	36	0.64	1,517,608	1,392,828	2,910,436	1,173,892	1,736,544	863,413
Vanuatu	VUT	Oceania	264,652	26	0.62	9,220	13,672	22,891	0	6,867	0
Venezuela	VEN	Andean	31,108,083	89	0.74	3,691,078	456,667	4,147,744	853,144	3,294,600	2,050,277
Viet Nam	VNM	Asia 2	91,703,800	34	0.59	4,107,474	4,258,583	8,366,058	2,091,514	6,274,543	3,764,726
Yemen	YEM	Middle East	26,832,215	35	0.46	1,238,074	1,227,039	2,465,113	456,046	2,009,067	1,269,533
Zambia	ZMB	SADC	16,211,767	41	0.43	312,933	1,294,269	1,607,202	321,440	1,285,761	803,601
Zimbabwe	ZWE	SADC	15,602,751	32	0.38	238,281	1,425,838	1,664,119	185,589	1,478,529	979,294

Notes: WB denotes World Bank; HDI denotes Human Development Index; Vax dogs denotes vaccinated dogs; Unvax dogs denotes unvaccinated dogs. [a] Democratic People's Republic of Korea. [b] Democratic Republic of the Congo. [c] Islamic Republic of Iran. [d] People's Democratic Republic.[e] Federated States of Micronesia. [f] Saint Vincent and the Grenadines. [g] The former Yugoslav Republic of Macedonia.[h] United Republic of Tanzania. [i] United States of America. [j] Bolivarian Republic of Venezuela. * CA & Mex. denotes Central America and Mexico. The specific numbers or estimates were derived from (1-6).

1.2 Vaccination costs

Table S2. Estimates of the costs of vaccination by dog rabies endemic country for the Global Dog Rabies Elimination Pathway (GDREP), 2017-2030, by phase of massive vaccine implementation

Country code	Cluster	NPV Phase I[a]	NPV Phase II[b]	NPV Phase III[c]	NPV total
AFG	Eurasia	1,415,799	8,250,414	23,141,085	32,807,298
ALB	EasternEurope	-	1,071,859	4,097,611	5,169,470
DZA	North Africa	-	8,813,392	25,888,349	34,701,740
AND	EU	-	-	-	-
AGO	Congo Basin	1,139,743	7,049,906	19,754,220	27,943,870
ATG	Caribbean	-	-	-	-
ARG	Southern Cone	-	17,750,433	51,811,992	69,562,424
ARM	EasternEurope	-	1,119,549	4,279,922	5,399,471
AUS	Oceania	-	-	-	-
AUT	EU	-	-	-	-
AZE	Eurasia	-	4,548,749	13,289,507	17,838,255
BHS	Caribbean	-	-	-	-
BHR	Middle East	-	535,394	1,555,817	2,091,210
BGD	Asia 3	10,836,721	42,744,548	120,873,135	174,454,405
BRB	Caribbean	-	-	-	-
BLR	EasternEurope	-	3,529,252	13,491,976	17,021,228
BEL	EU	-	-	-	-
BLZ	CA & Mex*	-	-	-	-
BEN	West Africa	514,002	3,066,346	8,597,191	12,177,539
BTN	Asia 3	74,260	209,845	599,303	883,408
BOL	Andean	-	-	13,985,241	13,985,241
BIH	EasternEurope	-	1,413,636	5,404,188	6,817,824
BWA	SADC	90,458	559,531	1,567,837	2,217,826
BRA	Brazil	-	41,611,672	263,143,254	304,754,926
BRN	Asia 1	-	-	-	-
BGR	EasternEurope	-	2,662,981	10,180,309	12,843,290
BFA	West Africa	965,271	5,758,453	16,145,118	22,868,843
BDI	Congo Basin	657,871	4,069,274	11,402,326	16,129,470
CIV	West Africa	972,462	5,801,353	16,265,398	23,039,214
CMR	Asia 2	618,918	3,781,884	10,599,150	14,999,953
CAN	West Africa	997,969	5,953,517	16,692,022	23,643,508
CSS	NorthAmerica	-	-	-	-
CPV	West Africa	-	-	-	-
CAF	Congo Basin	231,381	1,431,210	4,010,328	5,672,919
TCD	West Africa	793,061	4,731,111	13,264,732	18,788,903
CHL	Southern Cone	-	-	-	-
CHN	China	35,620,469	80,728,435	232,531,892	348,880,796

Country code	Cluster	NPV Phase I[a]	NPV Phase II[b]	NPV Phase III[c]	NPV total
COL	Andean	-	-	62,891,245	62,891,245
COM	SADC	-	-	-	-
COG	Congo Basin	160,830	1,049,243	2,937,570	4,147,643
COK	Oceania	-	-	-	-
CRI	CA & Mex*	-	-	-	-
HRV	EasternEurope	-	1,567,222	5,991,334	7,558,556
CUB	Caribbean	-	2,155,091	14,419,639	16,574,731
CYP	EasternEurope	-	-	-	-
CZE	EasternEurope	-	3,914,424	14,964,448	18,878,871
PRK	Asia 2	1,305,724	7,978,597	22,360,903	31,645,224
COD	Congo Basin	3,569,458	22,078,960	61,866,446	87,514,864
DNK	EU	-	-	-	-
DJI	North Africa	26,793	174,285	487,967	689,045
DMA	Caribbean	-	-	-	-
DOM	Caribbean	-	4,264,298	12,564,203	16,828,501
ECU	Andean	-	-	21,052,589	21,052,589
EGY	North Africa	-	27,099,404	79,601,456	106,700,860
SLV	CA & Mex*	-	1,176,138	7,756,498	8,932,637
GNQ	Congo Basin	39,942	247,062	692,282	979,286
ERI	North Africa	239,485	1,557,792	4,361,554	6,158,831
EST	EasternEurope	-	486,741	1,860,764	2,347,505
ETH	SADC	5,542,703	34,284,511	96,067,063	135,894,278
FJI	Oceania	-	-	-	-
FIN	EU	-	-	-	-
FRA	EU	-	-	-	-
GUF	Americas	-	-	-	-
GAB	Congo Basin	49,322	294,700	826,236	1,170,257
GMB	West Africa	80,609	480,884	1,348,267	1,909,760
GEO	Eurasia	-	1,733,939	5,065,830	6,799,769
DEU	EU	-	-	-	-
GHA	West Africa	1,175,782	7,014,283	19,666,119	27,856,184
GRC	EasternEurope	-	-	-	-
GRD	Caribbean	-	-	-	-
GTM	CA & Mex*	-	3,022,108	20,690,759	23,712,867
GIN	Congo Basin	610,425	3,775,800	10,579,996	14,966,221
GNB	West Africa	82,857	494,292	1,385,859	1,963,008
GUY	CA & Mex*	-	151,869	971,160	1,123,029
HTI	Caribbean	-	4,442,142	12,782,202	17,224,343
HND	CA & Mex*	-	-	10,530,048	10,530,048
HUN	EasternEurope	-	3,652,305	13,962,395	17,614,700
ISL	EU	-	-	-	-
IND	India	154,617,673	337,558,162	973,900,196	1,466,076,031
IDN	Indonesia	-	81,605,313	239,756,359	321,361,672

Country code	Cluster	NPV Phase I[a]	NPV Phase II[b]	NPV Phase III[c]	NPV total
IRN	Middle East	-	28,392,831	82,453,545	110,846,376
IRQ	Middle East	-	12,786,348	37,156,231	49,942,579
IRL	EU	-	-	-	-
ISR	Middle East	-	1,495,542	10,213,362	11,708,904
ITA	EU	-	-	-	-
JAM	Caribbean	-	-	-	-
JPN	Asia 1	-	-	-	-
JOR	Middle East	-	2,876,763	8,359,672	11,236,435
KAZ	Eurasia	-	5,572,981	16,281,878	21,854,860
KEN	SADC	2,450,347	15,156,676	42,469,829	60,076,851
KIR	Oceania	-	-	-	-
KWT	Middle East	-	1,585,754	4,608,090	6,193,844
KGZ	Eurasia	-	1,689,323	4,935,482	6,624,805
LAO	Asia 2	336,115	1,873,897	5,260,059	7,470,071
LVA	EasternEurope	-	733,986	2,805,957	3,539,943
LBN	Middle East	-	2,263,215	6,576,743	8,839,957
LSO	SADC	112,105	693,428	1,943,024	2,748,558
LBR	West Africa	201,602	1,202,683	3,371,992	4,776,276
LBY	North Africa	-	1,263,275	3,710,727	4,974,002
LTU	EasternEurope	-	1,079,662	4,127,440	5,207,102
LUX	EU	-	-	-	-
MDG	SADC	1,194,032	7,385,714	20,695,172	29,274,918
MWI	SADC	982,939	6,079,989	17,036,459	24,099,386
MYS	Asia 1	-	-	-	-
MDV	Asia 1	-	-	-	-
MLI	West Africa	862,055	5,142,706	14,418,732	20,423,493
MLT	EasternEurope	-	-	-	-
MHL	Oceania	-	-	-	-
MRT	West Africa	164,291	980,099	2,747,928	3,892,319
MUS	SADC	-	-	-	-
MEX	CA & Mex*	-	-	165,633,134	165,633,134
FSM	Oceania	-	-	-	-
MDA	EasternEurope	-	1,318,563	5,040,734	6,359,298
MCO	EU	-	-	-	-
MNG	Eurasia	444,848	971,183	2,801,991	4,218,021
MAR	North Africa	3,514,481	8,037,066	23,141,227	34,692,774
MOZ	SADC	3,032,571	8,569,429	24,473,727	36,075,727
MMR	Asia 2	2,359,965	14,420,511	40,415,080	57,195,556
NAM	SADC	109,331	676,270	1,894,946	2,680,548
NRU	Oceania	-	-	-	-
NPL	Asia 3	1,057,427	6,816,780	19,088,480	26,962,687
NLD	EU	-	-	-	-
NZL	Oceania	-	-	-	-
NIC	CA & Mex*	-	-	-	-

Country code	Cluster	NPV Phase I[a]	NPV Phase II[b]	NPV Phase III[c]	NPV total
NER	West Africa	1,156,271	6,897,887	19,339,778	27,393,936
NGA	West Africa	19,437,584	48,428,244	138,952,511	206,818,339
NIU	Oceania	-	-	-	-
NOR	EU	-	-	-	-
OMN	Middle East	695,802	1,519,060	4,382,690	6,597,551
PAK	Asia 3	13,110,810	51,714,504	146,238,398	211,063,712
PLW	Oceania	-	-	-	-
PAN	CA & Mex*	-	-	-	-
PNG	Oceania	-	-	-	-
PRY	Southern Cone	-	-	-	-
PER	Andean	-	13,098,397	37,443,788	50,542,185
PHL	Asia 4	10,022,355	28,321,142	80,883,322	119,226,820
POL	EasternEurope	-	8,039,137	55,510,239	63,549,376
PRT	EU	-	-	-	-
QAT	Middle East	-	914,682	2,658,002	3,572,684
KOR	Asia 1	-	-	-	-
ROU	EasternEurope	-	7,357,669	28,127,627	35,485,295
RUS	Eurasia	-	40,007,175	155,227,165	195,234,340
RWA	Congo Basin	-	3,865,242	11,408,339	15,273,581
KNA	Caribbean	-	-	-	-
LCA	Caribbean	-	-	-	-
VCT	Caribbean	-	-	-	-
WSM	Oceania	-	-	-	-
SMR	EU	-	-	-	-
STP	West Africa	-	-	-	-
SAU	Middle East	-	11,913,465	34,619,695	46,533,160
SEN	West Africa	716,253	4,272,903	11,980,044	16,969,201
SRB	EasternEurope	-	2,633,397	10,067,211	12,700,608
SYC	SADC	-	-	-	-
SLE	West Africa	316,013	1,885,217	5,285,629	7,486,859
SGP	Asia 1	-	-	-	-
SVK	EasternEurope	-	2,012,282	7,692,752	9,705,034
SVN	EasternEurope	-	765,643	2,926,974	3,692,617
SLB	Oceania	-	-	-	-
SOM	North Africa	486,806	3,166,554	8,865,815	12,519,175
ZAF	SADC	-	5,955,217	40,772,187	46,727,404
ESP	EU	-	-	-	-
LKA	Asia 4	-	4,201,703	15,765,538	19,967,241
SDN	North Africa	2,523,743	16,207,350	45,386,835	64,117,928
SUR	CA & Mex*	-	107,499	687,428	794,927
SWZ	SADC	70,788	437,860	1,226,908	1,735,557
SWE	EU	-	-	-	-
CHE	EU	-	-	-	-
SYR	Middle East	-	6,068,332	17,634,148	23,702,481

Country code	Cluster	NPV Phase I[a]	NPV Phase II[b]	NPV Phase III[c]	NPV total
TJK	Eurasia	946,371	2,094,009	6,037,912	9,078,293
THA	Asia 4	-	17,033,195	63,820,765	80,853,960
MKD	EasternEurope	-	771,091	2,947,802	3,718,892
TLS	Indonesia	-	-	-	-
TGO	West Africa	668,601	2,101,908	5,981,825	8,752,334
TON	Oceania	-	-	-	-
TTO	Caribbean	-	-	-	-
TUN	North Africa	-	2,070,707	7,816,936	9,887,643
TUR	EasternEurope	-	29,184,437	111,569,166	140,753,603
TKM	Eurasia	-	1,673,417	4,889,011	6,562,428
TUV	Oceania	-	-	-	-
UGA	SADC	4,792,285	13,542,026	38,675,137	57,009,449
UKR	EasternEurope	-	16,768,195	64,103,125	80,871,320
ARE	Middle East	-	3,501,892	10,176,252	13,678,143
GBR	EU	-	-	-	-
TZA	SADC	2,712,118	16,775,866	47,006,889	66,494,874
USA	NorthAmerica	-	-	-	-
URY	Southern Cone	-	-	-	-
UZB	Eurasia	-	6,910,949	26,830,545	33,741,494
VUT	Oceania	-	-	-	-
VEN	Andean	-	12,720,572	37,123,266	49,843,838
VNM	Asia 2	-	25,422,416	74,878,140	100,300,556
YEM	Middle East	-	7,592,520	22,063,330	29,655,850
ZMB	SADC	-	4,934,870	14,384,824	19,319,693
ZWE	SADC	1,798,437	4,761,507	13,630,346	20,190,290

Notes: NPV denotes net present value. Costs are in 2015 US dollars; we used a 3% discount rate (7). Estimates are based on a unit cost of $2.18 per dog vaccinated (point estimate) (8-10).

* CA & Mex. denotes Central America and Mexico

[a] Current costs of dog vaccination coverage plus 10% of the vaccination costs of the gap to reach the 70% goal.

[b] Costs of vaccination max (current vaccination coverage, median of the corresponding year in Phase II) plus 10% of the vaccination costs of the gap to reach the 70% goal.

[c] Costs of vaccination of 70% of the dog population.

1.3 Animal health workers vaccination capacity

Table S3. Animal health workers in public health by country and total dog vaccines needed to achieve dog rabies elimination following the Global Dog Rabies Elimination Pathway (GDREP), 2017-2030

Country code	Rabies cluster	WB Human Population 2015	Public Health animal workers	Public health para-veterinarians	Total dog vaccinations required.
AFG	Eurasia	32,526,562	96	48	17,636,799
ALB	EasternEurope	2,889,167	39	36	2,657,811
DZA	North Africa	39,666,519	1,356	35	17,969,523
AND	EU	70,473	-	-	-
AGO	Congo Basin	25,021,974	-	-	15,019,349
ATG	Caribbean	91,818	-	-	-
ARG	Southern Cone	43,416,755	1,833	1,151	35,963,545
ARM	EasternEurope	3,017,712	613	-	2,776,063
AUS	Oceania	23,781,169	-	-	-
AUT	EU	8,611,088	-	-	-
AZE	Eurasia	9,651,349	4	-	9,224,462
BHS	Caribbean	388,019	-	-	-
BHR	Middle East	1,377,237	34	31	1,079,918
BGD	Asia 3	160,995,642	528	478	93,931,031
BRB	Caribbean	284,215	-	-	-
BLR	EasternEurope	9,513,000	129	119	8,751,228
BEL	EU	11,285,721	-	-	-
BLZ	CA & Mex*	359,287	-	-	-
BEN	West Africa	10,879,829	14	13	6,545,984
BTN	Asia 3	774,830	82	77	476,510
BOL	Andean	10,724,705	407	239	7,006,807
BIH	EasternEurope	3,810,416	52	48	3,505,290
BWA	SADC	2,262,485	59	48	1,192,043
BRA	Brazil	207,847,528	5,609	3,614	154,874,121
BRN	Asia 1	423,188	-	-	-
BGR	EasternEurope	7,177,991	1,000	-	6,603,200
BFA	West Africa	18,105,570	403	400	12,293,050
BDI	Congo Basin	11,178,921	1,526	1,523	8,669,313
CIV	West Africa	22,701,556	408	315	12,384,632
CMR	Asia 2	15,577,899	-	-	8,062,532
CAN	West Africa	23,344,179	16	-	12,709,468
CSS	NorthAmerica	35,851,774	-	-	-
CPV	West Africa	520,502	-	-	-
CAF	Congo Basin	4,900,274	32	30	3,049,096
TCD	West Africa	14,037,472	411	200	10,099,895
CHL	Southern Cone	17,948,141	-	-	-

Country code	Rabies cluster	WB Human Population 2015	Public Health animal workers	Public health para-veterinarians	Total dog vaccinations required.
CHN	China	1,371,220,000	22,386	7,608	188,464,678
COL	Andean	48,228,704	262	-	31,509,420
COM	SADC	788,474	-	-	-
COG	Congo Basin	4,620,330	24	18	2,228,918
COK	Oceania	18,027	-	-	-
CRI	CA & Mex*	4,807,850	-	-	-
HRV	EasternEurope	4,224,404	492	-	3,886,127
CUB	Caribbean	11,389,562	831	260	8,422,883
CYP	EasternEurope	1,165,300	-	-	-
CZE	EasternEurope	10,551,219	419	-	9,706,310
PRK	Asia 2	25,155,317	567	390	17,009,428
COD	Congo Basin	77,266,814	235	128	47,037,734
DNK	EU	5,676,002	-	-	-
DJI	North Africa	887,861	20	15	370,292
DMA	Caribbean	72,680	-	-	-
DOM	Caribbean	10,528,391	130	10	8,727,282
ECU	Andean	16,144,363	630	320	10,547,650
EGY	North Africa	91,508,084	-	-	55,252,664
SLV	CA & Mex*	6,126,583	114	4	4,539,390
GNQ	Congo Basin	845,060	11	11	526,349
ERI	North Africa	5,222,000	54	4	3,309,747
EST	EasternEurope	1,311,998	54	7	1,206,937
ETH	SADC	99,390,750	2,494	2,159	73,040,836
FJI	Oceania	892,145	-	-	-
FIN	EU	5,482,013	-	-	-
FRA	EU	66,808,385	-	-	-
GUF	Americas	276,000	-	-	-
GAB	Congo Basin	1,725,292	23	22	629,064
GMB	West Africa	1,990,924	21	12	1,026,583
GEO	Eurasia	3,679,000	862	292	3,516,275
DEU	EU	81,413,145	-	-	-
GHA	West Africa	27,409,893	217	216	14,973,974
GRC	EasternEurope	10,823,732	-	-	-
GRD	Caribbean	106,825	-	-	-
GTM	CA & Mex*	16,342,897	55	10	12,050,163
GIN	Congo Basin	12,608,590	24	-	8,044,086
GNB	West Africa	1,844,325	13	11	1,055,207
GUY	CA & Mex*	767,085	14	0	570,711
HTI	Caribbean	10,711,067	27	15	8,946,019
HND	CA & Mex*	8,075,060	150	5	5,275,706
HUN	EasternEurope	9,844,686	656	204	9,056,354
ISL	EU	330,823	-	-	-

Country code	Rabies cluster	WB Human Population 2015	Public Health animal workers	Public health para-veterinarians	Total dog vaccinations required.
IND	India	1,311,050,527	21,403	7,274	792,189,639
IDN	Indonesia	257,563,815	1,270	-	166,418,784
IRN	Middle East	79,109,272	1,031	288	57,232,345
IRQ	Middle East	36,423,395	79	6	25,790,743
IRL	EU	4,640,703	-	-	-
ISR	Middle East	8,380,400	325	-	5,950,119
ITA	EU	60,802,085	-	-	-
JAM	Caribbean	2,725,941	-	-	-
JPN	Asia 1	126,958,472	-	-	-
JOR	Middle East	7,594,547	464	325	5,802,584
KAZ	Eurasia	17,544,126	1,041	123	11,301,516
KEN	SADC	46,050,302	1,207	1,103	32,290,274
KIR	Oceania	112,423	-	-	-
KWT	Middle East	3,892,115	40	-	3,198,550
KGZ	Eurasia	5,957,000	-	-	3,425,798
LAO	Asia 2	6,802,023	153	106	4,016,427
LVA	EasternEurope	1,978,440	188	-	1,820,013
LBN	Middle East	5,850,743	76	21	4,565,024
LSO	SADC	2,135,022	1	-	1,477,302
LBR	West Africa	4,503,438	48	28	2,567,467
LBY	North Africa	6,278,438	65	5	2,575,676
LTU	EasternEurope	2,910,199	533	-	2,677,159
LUX	EU	569,676	-	-	-
MDG	SADC	24,235,390	240	74	15,734,765
MWI	SADC	17,215,232	275	226	12,953,005
MYS	Asia 1	30,331,007	-	-	-
MDV	Asia 1	409,163	-	-	-
MLI	West Africa	17,599,694	438	-	10,978,562
MLT	EasternEurope	431,333	-	-	-
MHL	Oceania	52,993	-	-	-
MRT	West Africa	4,067,564	43	25	2,092,299
MUS	SADC	1,262,605	-	-	-
MEX	CA & Mex*	127,017,224	2,417	-	82,984,586
FSM	Oceania	104,460	-	-	-
MDA	EasternEurope	3,554,150	275	-	3,269,545
MCO	EU	37,731	-	-	-
MNG	Eurasia	2,959,134	295	-	2,279,195
MAR	North Africa	34,377,511	131	-	18,739,741
MOZ	SADC	27,977,863	446	367	19,459,216
MMR	Asia 2	53,897,154	36	-	30,742,829
NAM	SADC	2,458,830	84	73	1,440,748
NRU	Oceania	10,222	-	-	-
NPL	Asia 3	28,513,700	14	10	14,490,107

Country code	Rabies cluster	WB Human Population 2015	Public Health animal workers	Public health para-veterinarians	Total dog vaccinations required.
NLD	EU	16,936,520	-	-	-
NZL	Oceania	4,595,700	-	-	-
NIC	CA & Mex*	6,082,032	-	-	-
NER	West Africa	19,899,120	210	124	14,725,494
NGA	West Africa	182,201,962	1,454	758	111,648,510
NIU	Oceania	1,591	-	-	-
NOR	EU	5,195,921	-	-	-
OMN	Middle East	4,490,541	206	13	3,564,966
PAK	Asia 3	188,924,874	619	561	113,642,485
PLW	Oceania	21,291	-	-	-
PAN	CA & Mex*	3,929,141	-	-	-
PNG	Oceania	7,619,321	-	-	-
PRY	Southern Cone	6,639,123	-	-	-
PER	Andean	31,376,670	543	234	26,261,753
PHL	Asia 4	100,699,395	455	87	64,310,843
POL	EasternEurope	37,999,494	611	-	32,293,724
PRT	EU	10,348,648	-	-	-
QAT	Middle East	2,235,355	29	8	1,844,963
KOR	Asia 1	50,617,045	-	-	-
ROU	EasternEurope	19,832,389	2,086	1,149	18,244,272
RUS	Eurasia	144,096,812	8,547	1,008	100,187,439
RWA	Congo Basin	11,609,666	158	145	7,918,714
KNA	Caribbean	55,572	-	-	-
LCA	Caribbean	184,999	-	-	-
VCT	Caribbean	109,462	-	-	-
WSM	Oceania	193,228	-	-	-
SMR	EU	31,781	-	-	-
STP	West Africa	190,344	-	-	-
SAU	Middle East	31,540,372	160	-	24,030,093
SEN	West Africa	15,129,273	227	195	9,121,722
SRB	EasternEurope	7,098,247	160	-	6,529,841
SYC	SADC	92,900	-	-	-
SLE	West Africa	6,453,184	9	6	4,024,529
SGP	Asia 1	5,535,002	-	-	-
SVK	EasternEurope	5,424,050	722	25	4,989,709
SVN	EasternEurope	2,063,768	117	-	1,898,508
SLB	Oceania	583,591	-	-	-
SOM	North Africa	10,787,104	112	9	6,727,787
ZAF	SADC	54,956,920	111	84	23,745,455
ESP	EU	46,418,269	-	-	-
LKA	Asia 4	20,966,000	95	18	10,270,381
SDN	North Africa	52,574,694	547	43	34,458,235
SUR	CA & Mex*	542,975	15	14	403,973

Country code	Rabies cluster	WB Human Population 2015	Public Health animal workers	Public health para-veterinarians	Total dog vaccinations required.
SWZ	SADC	1,286,970	17	14	932,832
SWE	EU	9,798,871	-	-	-
CHE	EU	8,286,976	-	-	-
SYR	Middle East	18,502,413	241	67	12,240,149
TJK	Eurasia	8,481,855	503	59	4,904,938
THA	Asia 4	67,959,359	307	59	41,589,564
MKD	EasternEurope	2,078,453	68	-	1,912,017
TLS	Indonesia	1,245,015	-	-	-
TGO	West Africa	7,304,578	56	55	4,718,029
TON	Oceania	106,170	-	-	-
TTO	Caribbean	1,360,088	-	-	-
TUN	North Africa	11,107,800	339	94	5,085,097
TUR	EasternEurope	78,665,830	2,988	345	72,366,512
TKM	Eurasia	5,373,502	1,383	300	3,393,542
TUV	Oceania	9,916	-	-	-
UGA	SADC	39,032,383	198	100	30,750,847
UKR	EasternEurope	45,198,200	5,920	-	41,578,867
ARE	Middle East	9,156,963	32	-	7,063,502
GBR	EU	65,138,232	-	-	-
TZA	SADC	53,470,420	853	702	35,739,851
USA	NorthAmerica	321,418,820	-	-	-
URY	Southern Cone	3,431,555	-	-	-
UZB	Eurasia	31,299,500	2,784	-	17,317,095
VUT	Oceania	264,652	-	-	-
VEN	Andean	31,108,083	538	232	25,767,862
VNM	Asia 2	91,703,800	3,600	2,500	51,974,134
YEM	Middle East	26,832,215	350	98	15,314,516
ZMB	SADC	16,211,767	259	213	9,984,740
ZWE	SADC	15,602,751	182	133	10,895,107

Notes: *CA & Mex. denotes Central America and Mexico. Source of animal health worker data: World Organization for Animal Health (11); human population: World Bank (1).

Figure S1. Share of vaccination doses that the current animal public health workforce (11) could potentially deliver following the Global Dog Rabies Elimination Pathway (GDREP), by country

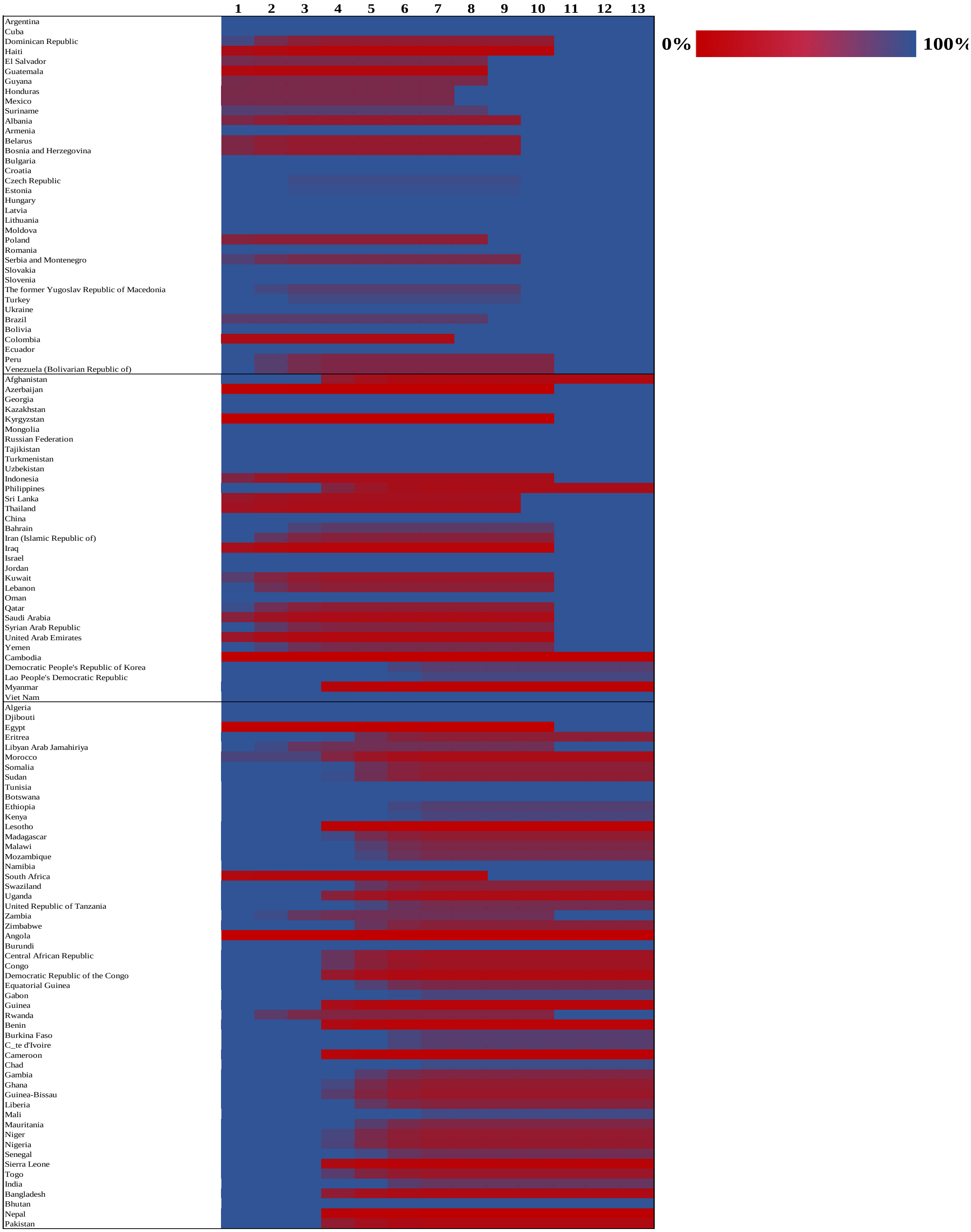

2 Appendix 2. Human development index and dog rabies elimination

Seventy countries were determined to be dog rabies virus free; 122 were categorized as DECs. The 70 DRFCs had a significantly higher mean HDI score of 0.78 compared to 0.60 for DECs ($p < 0.05$). Five mean HDI scores were calculated in accordance with the 13-year vaccination program phases and are displayed in table 3. Only 5 DECs were estimated to be vaccinating over 70% of dogs as of 2015; 56 (46%) were vaccinating less than 18% (Phase I). The remaining 61 DECs were defined as Phase II. The mean HDI score for DECs in Phase I was significantly lower than DECs in Phase II and Phase III (mean 0.46 vs 0.71, $p = 0.004$). Figure S2 shows the association.

Figure S2. Association between human development index (HDI) and dog vaccination coverage.

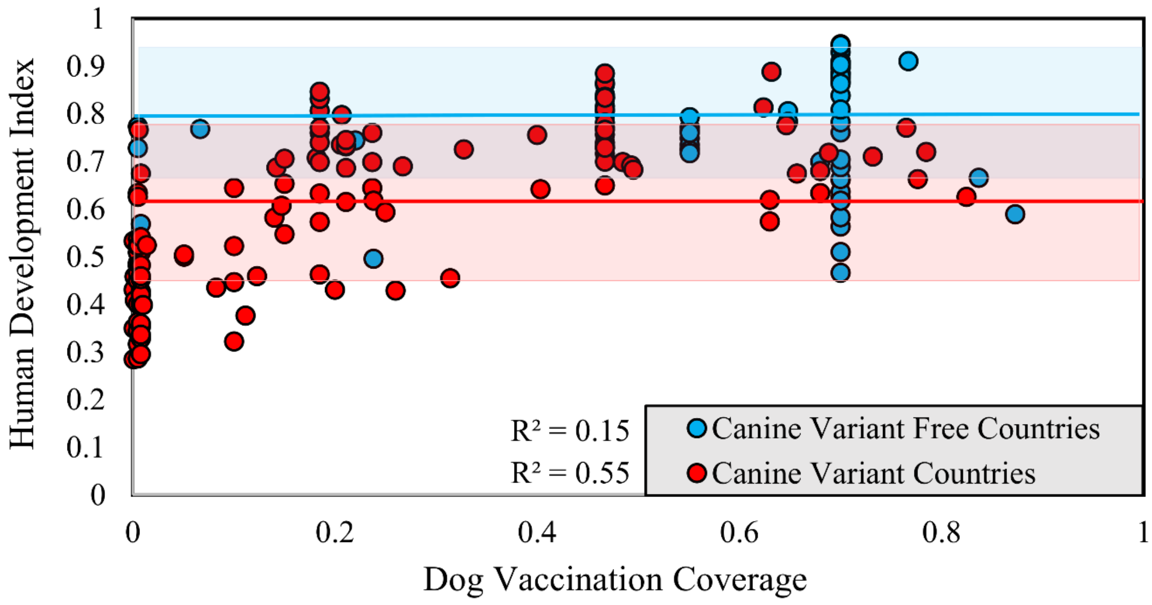

3 References for the supplementary material

1. World Bank. World Bank Open Data (2016) [cited 2016 October 10]. Available from: http://data.worldbank.org/indicator/SP.URB.TOTL.IN.ZS.

2. Knobel DL, Cleaveland S, Coleman PG, Fèvre EM, Meltzer MI, Miranda MEG, et al. Re-evaluating the burden of rabies in Africa and Asia. *Bull WHO* (2005) **83**(5):360-8.

3. Hampson K, Coudeville L, Lembo T, Sambo M, Kieffer A, Attlan M, et al. Estimating the Global Burden of Endemic Canine Rabies. *PLoS Negl Trop Dis* (2015) **9**(4):e0003709.

4. Coleman PG, Dye C. Immunization coverage required to prevent outbreaks of dog rabies. *Vaccine* (1996) **14**(3):185-6.

5. Cleaveland S, Kaare M, Tiringa P, Mlengeya T, Barrat J. A dog rabies vaccination campaign in rural Africa: impact on the incidence of dog rabies and human dog-bite injuries. *Vaccine* (2003) **21**(17):1965-73.

6. World Health Organization. WHO Expert Consultation on Rabies Geneva(2013) [cited 2016 30 March]. Available from: http://apps.who.int/iris/bitstream/10665/85346/1/9789240690943_eng.pdf.

7. World Health Organization. WHO-CHOICE. Choosing interventions that are cost-effective Geneva: WHO_CHOICE (2015) [cited 2015 November 20]. Available from: http://www.who.int/choice/en/.

8. Kayali U, Mindekem R, Hutton G, Ndoutamia A, Zinsstag J. Cost-description of a pilot parenteral vaccination campaign against rabies in dogs in N'Djaména, Chad. *Trop Med Int Health* (2006) **11**(7):1058-65.

9. Kaare M, Lembo T, Hampson K, Ernest E, Estes A, Mentzel C, et al. Rabies control in rural Africa: evaluating strategies for effective domestic dog vaccination. *Vaccine* (2009) **27**(1):152-60.

10. Lapiz SMD, Miranda MEG, Garcia RG, Daguro LI, Paman MD, Madrinan FP, et al. Implementation of an intersectoral program to eliminate human and canine rabies: the Bohol Rabies Prevention and Elimination Project. *PLoS Negl Trop Dis* (2012) **6**(12):e1891.

11. World Organization for Animal Health (OIE). World Animal Health Information Database (WAHIS) Interface (2016) [cited 2016 October 1]. Available from: http://www.oie.int/wahis_2/public/wahid.php/Wahidhome/Home.
